# Antimicrobial and antioxidant activities of triterpenoid and phenolic derivatives from two Cameroonian Melastomataceae plants: *Dissotis senegambiensis* and *Amphiblemma monticola*

**DOI:** 10.1186/s12906-018-2229-2

**Published:** 2018-05-16

**Authors:** Raissa Tioyem Nzogong, Fabrice Sterling Tchantchou Ndjateu, Steve Endeguele Ekom, Jules-Arnaud Mboutchom Fosso, Maurice Ducret Awouafack, Mathieu Tene, Pierre Tane, Hiroyuki Morita, Muhammad Iqbal Choudhary, Jean-de-Dieu Tamokou

**Affiliations:** 1 Laboratory of Natural Products Chemistry, Department of Chemistry, Faculty of Science, University of Dschang, P.O. Box 67, Dschang, Cameroon; 2 Laboratory of Microbiology and Antimicrobial substances, Department of Biochemistry, Faculty of Science, University of Dschang, P.O. Box 67, Dschang, Cameroon; 3 Institute of Natural Medicine, University of Toyama, 2630-Sugitani, Toyama, 930-0194 Japan; 4 H.E.J Research Institute of Chemistry, University of Karachi, -75270, Karachi, Pakistan

**Keywords:** *Dissotis senegambiensis*, *Amphiblemma monticola*, Melastomataceae, Triterpenoids, Phenolics, Antibacterial, Antifungal, Methicillin-resistant *S. aureus*

## Abstract

**Background:**

Antimicrobial resistance is a serious threat against humankind and the search for new therapeutics is needed. This study aims to investigate the antimicrobial and antioxidant activities of ethanol extracts and compounds isolated from *Dissotis senegambiensis* and *Amphiblemma monticola*, two Cameroonian Melastomataceae species traditionally used for the treatment of fever, malaria and infectious diseases.

**Methods:**

The plant extracts were prepared by maceration in ethanol. Standard chromatographic and spectroscopic methods were used to isolate and identify fourteen compounds from the two plant species [**1–6** (from *D. senegambiensis*), **3**, **4** and **7–14** (from *A. monticola*)]. A two-fold serial micro-dilution method was used to determine the minimum inhibitory concentration (MIC) against four bacterial strains including two resistant bacterial strains, methicillin resistant *S. aureus* (MRSA3) and methicillin resistant *S. aureus* (MRSA4) and three yeast strains.

**Results:**

The fractionation of EtOH extracts afforded fourteen compounds belonging to triterpenoid and phenolic derivatives. The ethanol extracts, compounds **3**, **5–8, 10** and the mixture of **10** + **12** were active against all the tested bacterial and fungal species. Compound **7** (MIC = 16–32 μg/mL) and **10** (MIC = 8–16 μg/mL) displayed the largest antibacterial and antifungal activities, respectively. Compounds **7**, **10** and the mixture of **10** + **12** showed prominent antibacterial activity against methicillin- resistant *S. aureus* (MRSA) which is in some cases equal to that of ciprofloxacin used as reference antibacterial drug. Compound **8** also showed high radical-scavenging activities and ferric reducing power when compared with vitamin C and butylated hydroxytoluene used as reference antioxidants. The tested samples were non-toxic to normal cells highlighting their good selectivity.

**Conclusions:**

The result of this investigation reveals the potential of *D. senegambiensis* and *A. monticola* as well as the most active compounds in the search for new antimicrobial and antioxidant agents. So, further investigations are needed.

**Electronic supplementary material:**

The online version of this article (10.1186/s12906-018-2229-2) contains supplementary material, which is available to authorized users.

## Background

Infectious diseases are among the leading causes of death accounting for approximately one-half of all deaths in developing countries [1]. Despite the successes of the Millennium Development Goals era, the inhabitants of low-income countries still suffer an enormous burden of disease owing to diarrhoea, pneumonia, HIV/AIDS, tuberculosis, malaria and other infectious diseases. Increase in infections as a result of emergence of drug-resistant microorganisms and hitherto unknown pathogenic microbes pose enormous public health concerns [1]. These therefore, necessitate continued search for compounds with antimicrobial activities. Historically, plants have provided a good source of anti-infective agents in the fight against microbial infections [2–5]. The genus *Dissotis* which belongs to the Melastomataceae family comprises about 140 species in Africa [6]. They are climbing shrubs, shrubs or small trees found in some African countries such as Ivory Cost, Benin, Democratic Republic of Congo, Nigeria and Cameroon [7]. Several species are used in folk medicine as antidiarrheic, antimicrobial, antioxidant, antitumoral, anti-rheumatic, and anti-inflammatory agents, and also in the treatment of skin diseases, fever, malaria, and to lower blood cholesterol [8]. *Dissotis senegambiensis* (Guill. & Perr.) Triana (Syn. *Dissotis irvingiana* Hook) belonging to the Melastomataceae family, is a shrub reaching 120 cm in height. The flowers are purple. In Africa, this plant species is found in tropical areas of Cameroon, Senegal, Ethiopia and Mozambique [7]. This species is used in traditional medicine for the treatment of the kwashiorkor, anemia, marasmus, avitaminose, drepanocytose, cutaneous eruptions and diarrhea [9]. To the best of our knowledge, no phytochemical work has yet been done on *D. senegambiensis*. The genus *Amphiblemma* belonging also to the Melastomataceae family, extends from tropical West Africa to Ethiopia and Cabinda. It contains at least 14 species distributed in Africa [10]. They are herbaceous plants or shrubs that grow in evergreen forests [10]. *Amphiblemma monticola* Jacq.-Fél. is a prostrate herb or sub-shrub reaching 100 cm in height that generally grows in West and South-West Regions of Cameroon [10–12]. This plant species is used by the Bamena populations in West Region of Cameroon against fever and stomach disorders [13]. Previous phytochemical studies of some species of the Melastomataceae family reported the isolation of terpenoids, steroids, simple phenolics, flavonoids and a vast range of polyphenols [14–18]. According to some traditional healers found in the Western region of Cameroon, maceration of the studied plants in raffia wine (a traditional alcoholic beverage produced in several African countries) is used for the treatment of different diseases. Traditional uses of *D. senegambiensis* and *A. monticola* motivated our effort to investigate the phytochemistry and pharmacological activity. Fourteen compounds [*β*-amyrin palmitate (**1**), *α*-amyrin acetate (**2**), ursolic acid (**3**), sitosterol-3-O-*β*-D-glucopyranoside (**4**), vitexin (**5**) and *trans*-tiliroside (**6**) (from *D. senegambiensis*), ursolic acid (**3**), sitosterol-3-O-*β*-D-glucopyranoside (**4**), 3,4′-di-*O*-methylellagic acid (**7**), dimethyl 4,4′,5,5′,6,6′-hexahydroxybiphenyl-2,2′-dicarboxylate (**8**), lupeol (**9**), ellagic acid (**10**), 3-hydroxy-4,5-dimethoxybenzoic acid (**11**), 3-*O*-methylellagic acid 4′-*O*-*β*-D-xylopyranoside (**12**), oleanolic acid (**13**) and amphiblemmone A (**14**) (from *A. monticola*)] were isolated and characterized. This is the first report on the isolation of compounds **1–6** from *D. senegambiensis*. Compounds **3**, **4** and **7–14** were previously isolated from the same source (*A. monticola*) [13]. Antimicrobial and antioxidant activities of ethanol extracts of *D. senegambiensis* and *A. monticola* and some compounds (**3–10**, a mixture of **3** and **13**, and a mixture of **10** and **12**) isolated in sufficient quantities are reported here for the first time.

## Methods

### General experimental procedures

MS data were measured on JEOL MS Station JMS-700 spectrometer or JEOL 600 MS Route spectrometer. ^1^H NMR (500 and 400 MHz) and ^13^C NMR (125 and 100 MHz) were recorded using JEOL spectrometers or Bruker Avance AV-400 spectrometer. The chemical shifts were reported in parts per million (ppm) with TMS as internal standard. Deuterated solvents, methanol (CD_3_OD), dimethyl sulfoxide (DMSO-*d*_*6*_), pyridine (C_5_D_5_N) and chloroform (CDCl_3_) were used as solvents for the NMR experiments. CC was performed on silica gel 60 F_254_ (70–230 mesh; Merck) and gel permeation on Sephadex LH-20. TLC was carried out on precoated silica gel Kieselgel 60 F_254_ plates (0.25 mm thick), and spots weredetected with UV lights (254 and 365 nm) and further sprayedwith 20% H_2_SO_4_ reagent followed by heating to 100 °C.

### Sample collections

Plant materials were collected in two locations of the Western Region of Cameroon: the whole plant of *Dissotis senegambiensis* (Guill. & Perr.) Triana in Bansoa (January 2013) and the roots of *Amphiblemma monticola* Jacq.-Fél. in Bamena (May 2016). Their identification was done by Mr. Fulbert Tadjouteu, a botanist of the Cameroon National Herbarium in Yaoundé, where voucher specimens, N^o^ 24736/SRF/Cam (*D. senegambiensis*) and N^o^ 45094/HNC (*A. monticola*), were deposited.

### Extraction

The powdered material of *D. senegambiensis* (1.8 kg) was extracted three times (72 h for each time) by maceration with ethanol (8 L) at room temperature. Evaporation of solvent under vacuum afforded 78 g of crude extract. A portion of this extract (76 g) was successively triturated with *n*-hexane, EtOAc and *n*-butanol. TLC analysis showed that the *n-*hexane and EtOAc extracts (19.5 and 20.5 g, respectively) were qualitatively the same. They were thus combined to afford 40 g of extract called “EtOAc extract”.

Dried and pulverized roots (1.5 kg) and aerial part (0.08 kg) of *A. monticola* were respectively macerated with ethanol (5 L with roots and 1 L with aerial part) for 24 h (3 times) at room temperature. Evaporation of solvent under reduced pressure afforded 49 g and 4.28 g of crude extracts, respectively.

### Phytochemical analysis

The extracts were screened for secondary metabolites using standard procedures as previously described [19–22]. The plant extracts were screened for the presence of different classes of compounds including triterpenoids, steroids, flavonoids, phenols, glycosides, tannins and alkaloids.

### Isolation of constituents

A portion (38 g) of “EtOAc extract” of *D. senegambiensis* was subjected to silica gel (70 to 230 mesh) column chromatography (CC) eluted with gradient of *n*-hexane-EtOAc (100:0, 9:1, 4:1, 7:3, 3:2, 1:1 and 0:100) followed by gradient of EtOAc-MeOH (19:1, 9:1, 4:1, 7:3, 1:1 and 0:100). Fifty-five fractions of 300 mL each were collected and combined into six major fractions on the basis of their TLC profiles: A (1–6; 4.0 g), B (7–12; 4.5 g), C (13–17; 3.6 g), D (18–26; 4.7 g), E (27–36; 5.5 g), and F (37–55; 9.1 g). Fraction A crystallized to afford a mixture of two compounds. This mixture was subjected to silica gel CC and eluted with *n*-hexane- EtOAc (49:1) to yield *β*-amyrin palmitate (4.2 mg; **1**) and *α*-amyrin acetate (3.5 mg; **2**). Fraction C crystallized to afford ursolic acid (15.0 mg; **3**). Fraction E was subjected to silica gel CC and eluted with CH_2_Cl_2_ –MeOH mixture of increasing polarity to yield sitosterol-3-*O*-*β*-D-glucopyranoside (35.1 mg; **4**) and vitexin (28.5 mg; **5**). Similarly as with fraction E, fraction F afforded *trans*-tiliroside (25.0 mg; **6**). A portion (18 g) of the *n*-BuOH extract was also subjected to silica gel CC eluted with gradient of CH_2_Cl_2_-MeOH (100:0, 19:1, 9:1, 4:1and 0:100). Twenty-two fractions of 300 mL each were collected and combined into four major fractions on the basis of their TLC profiles: G (1–7; 2.7 g), H (8–12; 3.5 g), I (13–18; 3.6 g) and J (19–22; 3.7 g). Fraction G was subjected to silica gel CC and eluted with CH_2_Cl_2_ –MeOH mixture of increasing polarity to yield vitexin (15.1 mg; **5**) and *trans*-tiliroside (13.1 mg; **6**). An attempt to purify fractions B, D, H, I and J failed.

A portion (47 g) of EtOH extract of the roots of *A. monticola* was fractionated on silica gel CC eluted with CH_2_Cl_2_-MeOH of increasing polarity to give 25 fractions of 300 mL each. After comparative TLC, they were combined into 4 major fractions: A (1–8; 7.6 g), B (9–16; 11 g), C (17–21; 5.1 g) and D (22–25; 5.8 g). Fraction A was chromatographed on a silica gel column eluted with a continuous gradient of *n*-hexane-EtOAc to afford lupeol (**9**, 120.8 mg) and a mixture of sterols. Similarly, fractions B and C were eluted with CH_2_Cl_2_-MeOH of increasing polarity yielding four (B1-B4) and three (C1-C3) sub-fractions, respectively. B2 (1.9 g), B3 (2.3 g), C2 (1.9 g) and C3 (1.2 g) were passed separately on LH-20 Sephadex CC eluted with CH_2_Cl_2_-MeOH (1:1) to give 3,4′-di-*O*-methylellagic acid (20.0 mg; **7**) from B2, dimethyl 4,4′,5,5′,6,6′-hexahydroxybiphenyl-2,2′-dicarboxylate (15.0 mg; **8**) from B3, ellagic acid (23.0 mg; **10**), 3-hydroxy-4,5-dimethoxybenzoic acid (4.0 mg; **11**) and a mixture of **10** and **12** (7.0 mg) from C2, and 3-*O*-methylellagic acid 4′-*O*-*β*-D-xylopyranoside (2.3 mg; **12**) from C3. Re-crystallization of B4 (0.7 g) in EtOAc afforded a mixture (31.9 mg) of ursolic acid (**3**) and oleanolic acid (**13**). Fraction D was subjected to silica gel CC eluted with a gradient mixture of CH_2_Cl_2_-MeOH to afford four sub-fractions (D1-D4). Repeated silica gel CC of D2 (0.8 g), eluted with CH_2_Cl_2_-MeOH (from 49:1 to 9:1) gave sitosterol-3-O-*β*-D-glucopyranoside (45.0 mg; **4**) and amphiblemmone A (9.7 mg; **14**).

Due to the small quantity of plant material, the aerial part of *A. monticola* (4.28 g of crude EtOH extract), compared to the roots (same collection in the field), was not further studied in this work.

### Antimicrobial activity of extracts and compounds

#### Tested microorganisms

The microorganisms used in this study include four bacterial (*Staphylococcus aureus* ATCC25923, methicillin sensitive *S. aureus* MSSA1, methicillin resistant *S. aureus* MRSA3 and methicillin resistant *S. aureus* MRSA4) and three yeast strains (*Candida albicans* ATCC10231, *Candida tropicalis* PK233 and *Cryptococcus neoformans* H99). These microorganisms were taken from our laboratory collection. The fungal and bacterial strains were grown at 37 °C and maintained on Sabouraud Dextrose Agar (SDA, Conda, Madrid, Spain) and nutrient agar (NA, Conda) slants respectively.

### Inocula preparation

The inocula of bacteria and yeasts were prepared from overnight cultures as previously described [23]. Absorbance was read spectrophotometrically at 530 nm and 600 nm for yeasts and bacteria respectively. The final concentrations of microbial suspensions were 2.5 × 10^5^ cells/mL for yeasts and 10^6^ CFU/mL for bacteria.

### Antimicrobial assay

The antimicrobial activity was evaluated by determining the minimum inhibitory concentrations (MICs). MICs of extracts and compounds were determined by broth micro dilution [24]. Each test sample was dissolved in 10% *v*/v aqueous dimethylsulfoxide (DMSO) to give a stock solution. This was serially diluted two-fold in Mueller-Hinton Broth (MHB) for bacteria and Sabouraud Dextrose Broth (SDB) for fungi to obtain a concentration range of 4096 to 0.25 μg/mL. Then, 100 μL of each sample concentration was added to respective wells (96-well micro plate) containing 90 μL of SDB/ MHB and 10 μL of inoculum to give final concentration ranges of 2048 to 4 μg/mL (for extracts) and 256 to 0.125 μg/mL (for compounds). Dilutions of nystatin (Sigma-Aldrich, Steinheim, Germany) and ciprofloxacin (Sigma-Aldrich, Steinheim, Germany) were used as positive controls for yeasts and bacteria respectively. Broth with 10 μL of DMSO was used as negative control. The cultured micro plates were covered; then, the contents of each well were mixed thoroughly using a plate shaker (Flow Laboratory, Germany) and incubated at 37 °C for 24 h (bacteria) and 48 h (yeasts) under shaking. After the incubation period, MICs were assessed visually and were taken as the lowest sample concentration at which there was no growth or virtually no growth. The lowest concentration that yielded no growth after the sub-culturing was considered as the minimum microbicidal concentrations (MMCs). All the tests were performed in triplicate.

### Antioxidant assay

#### Ferric reducing antioxidant power (FRAP) assay

The FRAP was determined by the Fe^3+^-Fe^2+^ transformation in the presence of extracts and compounds as previously described [25]. The Fe^2+^ was monitored by measuring the formation of Perl’s Prussian blue at 700 nm. Butylated hydroxytoluene (BHT) was used as a positive control. All the tests were performed in triplicate.

### Diphenyl-1-picrylhydrazyl (DPPH) free radical scavenging assay

The free radical scavenging activity of extracts and compounds was evaluated according to described methods [26]. The EC_50_ (μg/ml), which is the amount of sample necessary to inhibit by 50% the absorbance of free radical DPPH was calculated [26]. Vitamin C was used as a standard control. All the analyses were carried out in triplicate.

### Hemolytic assay

Whole blood (10 mL) from albino rats was collected by cardiac puncture into a conical tube containing EDTA as an anticoagulant. The study was conducted according to the ethical guidelines of the Committee for Control and Supervision of Experiments on Animals (Registration no. 173/CPCSEA, dated 28 January, 2000), Government of India, on the use of animals for scientific research. Erythrocytes were harvested by centrifugation at room temperature for 10 min at 1000 x *g* and were washed three times in PBS buffer [27]. The cytotoxicity was evaluated as previously described [27].

### Statistical analysis

Data were analyzed by one-way analysis of variance followed by Waller-Duncan Post Hoc test. The experimental results were expressed as the mean ± Standard Deviation (SD). Differences between groups were considered significant when *p* < 0.05. All analyses were performed using the Statistical Package for Social Sciences (SPSS, version 12.0) software.

## Results

### Chemical analysis

**Fig. 1:**
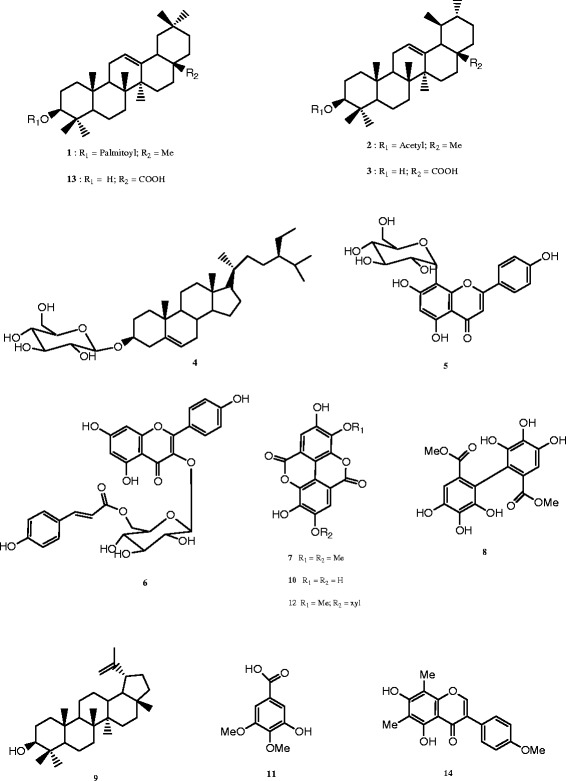
Chemical structures of compounds isolated from *D. senegambiensis* (**1–6**) and *A. monticola* (**3**, **4**, **7–14**). **1**: *β*-amyrin palmitate; **2**: *α*-amyrin acetate; **3**: ursolic acid; **4**: sitosterol 3-*O-β*-D-glucopyranoside; **5**: vitexin; **6**: *trans*-tilliroside; **7**: 3,4′-di-*O*-methylellagic acid; **8**: dimethyl 4,4′,5,5′,6,6′-hexahydroxybiphenyl-2,2′-dicarboxylate; **9**: lupeol; **10**: ellagic acid; **11**: 3-hydroxy-4,5-dimethoxybenzoic acid; **12**: 3-*O*-methylellagic acid 4′-*O*-*β*-D-xylopyranoside; **13**: oleanolic acid; **14**: amphiblemmone A

The phytochemical screening revealed the presence of steroids, phenols, glycosides and tannins in all the plant extracts (Table 1). Triterpenoids and flavonoids are selectively distributed in the extracts whereas alkaloids were absent in all the extracts (Table 1). The EtOAc and *n*-BuOH extracts from *D. senegambiensis* and EtOH extract from the roots of *A. monticola* were fractionated by silica gel column chromatography to afford fourteen compounds (**1**–**14**) (Fig. 1). Compounds obtained from *D. senegambiensis* were identified as *β*-amyrin palmitate (**1**) [28], *α*-amyrin acetate (**2**) [29], ursolic acid (**3**) [30], sitosterol-3-O-*β*-D-glucopyranoside (**4**) [31]; vitexin (**5**) [32] and *trans*-tiliroside (**6**) [33]. From *A. monticola*, compounds were identified as 3,4′-di-*O*-methylellagic acid (**7**) [34], dimethyl 4,4′,5,5′,6,6′-hexahydroxybiphenyl-2,2′-dicarboxylate (**8**) [35], lupeol (**9**) [36], ellagic acid (**10**) [16], 3-hydroxy-4,5-dimethoxybenzoic acid (**11**) [37], 3-*O*-methylellagic acid 4′-*O*-*β*-D-xylopyranoside (**12**) [38], oleanolic acid (**13**) [16], and amphiblemmone A (**14**) [13]. The structures of the compounds (Fig. 1) were determined by analysis of their NMR data and comparison with those reported in the literature (Additional file 1).

**Table 1:** Secondary metabolites identified in the studied plant extracts

Metabolites	*D. senegambiensis*			*A. monticola*	
	Whole plant			Roots	Aerial part
	Crude EtOH extract	EtOAc extract	*n*-BuOH extract	Crude EtOH extract	Crude EtOH extract
Triterpenoids	+	+	–	+	+
Steroids	+	+	+	+	+
Flavonoids	+	+	+	+	–
phenols	+	+	+	+	+
Tannins	+	+	+	+	+
Glycosides	+	+	+	+	+
Alkaloids	–	–	–	–	–

(+): presence; (−): absence

### Antimicrobial activity

The antimicrobial activity of EtOH extracts from *D. senegambiensis* and *A. monticola* as well as their isolated compounds was performed against four bacterial strains including two resistant bacterial strains, methicillin resistant *S. aureus* (MRSA3) and methicillin resistant *S. aureus* (MRSA4) and three yeast strains (Table 2). The EtOH, EtOAc and *n*-BuOH extracts, as well as compounds **3**, **5–8, 10** and the mixture of **10** + **12** were active against all the tested bacterial and fungal species. Among the extracts, the EtOH extract from *D. senegambiensis* (MIC = 64–256 μg/mL) was the most active against *S. aureus* strains whereas the *A. monticola* EtOH extract (MIC = 128–256 μg/mL) was the most effective against yeast strains. The results also showed that *S. aureus* ATCC25923 and *S. aureus* MSSA1 were the most sensitive bacteria while the most sensitive fungi were *C. tropicalis* and *C. neoformans*. Compound **10** (MIC = 8–16 μg/mL) displayed the largest antifungal activity whereas compound **7** (MIC = 16–32 μg/ml) showed the best anti-staphylococcal activity. Compound **10** (MIC = 8–32 μg/mL) was the most active sample against bacterial and fungal strains following in decreasing order by **7** (MIC = 16–32 μg/mL), **10 + 12** (MIC = 16–64 μg/mL), **8** (MIC = 8–128 μg/mL), **6** (MIC = 32–128 μg/mL), **5** (MIC = 64–128 μg/mL), **3** (MIC = 128–256 μg/mL), **3 + 13** (MIC = 64 - > 256 μg/mL), **9** (MIC = 256 - > 256 μg/mL) and **4** (MIC = 128 - > 256 μg/mL). Compounds **1** and **2**, obtained in small quantities, were not tested against the microorganisms used. The standard drugs used in this study were ciprofloxacin and nystatin for antibacterial and antifungal activity, respectively, and the antibacterial activities of some of the isolated compounds are in some cases equal to those of ciprofloxacin whereas the antifungal activity of the isolated compounds is lesser than that of nystatin.

**Table 2:** Antimicrobial activity (in μg/ml) of extracts and isolated compounds from *D. senegambiensis* and *A. monticola* against bacterial and yeast strains

Crude extracts/compounds	Inhibition parameters	*S. aureus* ATCC25923	*S. aureus* MSSA1	*S. aureus* MRSA3	*S. aureus* MRSA4	*C. albicans* ATCC10231	*C. tropicalis* PK233	*C. neoformans* H99
DSEtOH	MIC	128	64	256	128	2048	1024	512
	MMC	256	128	512	256	2048	2048	1024
	MMC/MIC	2	2	2	2	1	2	2
DSEtOAc	MIC	256	128	256	256	2048	1024	512
	MMC	256	256	512	512	> 2048	> 2048	> 2048
	MMC/MIC	1	2	2	1	nd	nd	nd
DSBuOH	MIC	256	64	256	128	2048	1024	1024
	MMC	512	128	512	256	2048	> 2048	> 2048
	MMC/MIC	2	2	2	2	1	nd	nd
AMEtOH	MIC	256	128	256	256	256	128	256
	MMC	256	128	512	512	512	256	256
	MMC/MIC	1	1	2	2	2	2	1
AMEtOAc	MIC	512	256	512	512	512	2048	2048
	MMC	512	512	512	512	1024	> 2048	> 2048
	MMC/MIC	1	2	1	1	2	nd	nd
3	MIC	256	128	128	128	256	256	128
	MMC	> 256	> 256	> 256	> 256	> 256	> 256	> 256
	MMC/MIC	nd	nd	Nd	nd	nd	nd	nd
4	MIC	> 256	> 256	> 256	> 256	256	256	128
	MMC	> 256	> 256	> 256	> 256	> 256	> 256	> 256
	MMC/MIC	nd	nd	Nd	nd	nd	nd	nd
5	MIC	64	64	64	128	128	64	128
	MMC	128	128	128	256	256	128	128
	MMC/MIC	2	2	2	2	2	2	1
6	MIC	32	64	64	128	64	64	64
	MMC	64	64	128	128	64	64	128
	MMC/MIC	2	1	2	1	1	1	2
7	MIC	32	16	16	32	32	32	32
	MMC	32	16	32	64	64	64	32
	MMC/MIC	1	1	2	2	2	2	1
8	MIC	32	32	64	128	128	16	32
	MMC	64	32	128	256	> 256	16	32
	MMC/MIC	2	1	2	2	nd	1	1
9	MIC	256	256	> 256	> 256	> 256	256	256
	MMC	> 256	> 256	> 256	> 256	nd	> 256	> 256
	MMC/MIC	nd	nd	Nd	nd	nd	nd	nd
10	MIC	8	16	32	32	16	8	16
	MMC	16	16	64	32	16	8	16
	MMC/MIC	2	1	2	1	1	1	1
10 + 12	MIC	32	16	32	32	64	64	64
	MMC	64	64	64	64	128	128	64
	MMC/MIC	2	4	2	2	2	2	1
3 + 13	MIC	128	64	> 256	128	> 256	> 256	128
	MMC	256	128	Nd	256	> 256	> 256	256
	MMC/MIC	2	2	nd	2	nd	nd	2
Reference drugs*	MIC	1	1	16	32	2	0.5	1
	MMC	1	1	16	32	2	1	1
	MMC/MIC	1	1	1	1	1	2	1

*: Ciprofloxacin for bacteria and nystatin for fungi; compounds **1–6** and compounds **3, 4, 7–14** were isolated from *D. senegambiensis* and *A. monticola* respectively; compounds **1–2**, **11** and **14** were not tested; nd: not determined. MIC: Minimum Inhibitory Concentrations; MMC: Minimum Microbicidal Concentrations; DSEtOH = *D. senegambiensis* EtOH extract; DSEtOAc = *D. senegambiensis* EtOAc extract; DSBuOH = *D. senegambiensis n*-BuOH extract; AMEtOH = *A. monticola* EtOH extract; AMEtOAc = *A. monticola* EtOAc extract; **3**: ursolic acid; **4**: sitosterol 3-*O-β*-D-glucopyranoside; **5**: vitexin; **6**: *trans*-tilliroside; **7**: 3,4′-di-*O*-methylellagic acid; **8**: dimethyl 4,4′,5,5′,6,6′-hexahydroxybiphenyl-2,2′-dicarboxylate; **9**: lupeol; **10**: ellagic acid; **12**: 3-*O*-methylellagic acid 4′-*O*-*β*-D-xylopyranoside; **13**: oleanolic acid

### Ferric reducing antioxidant power (FRAP)

**Fig. 2:**
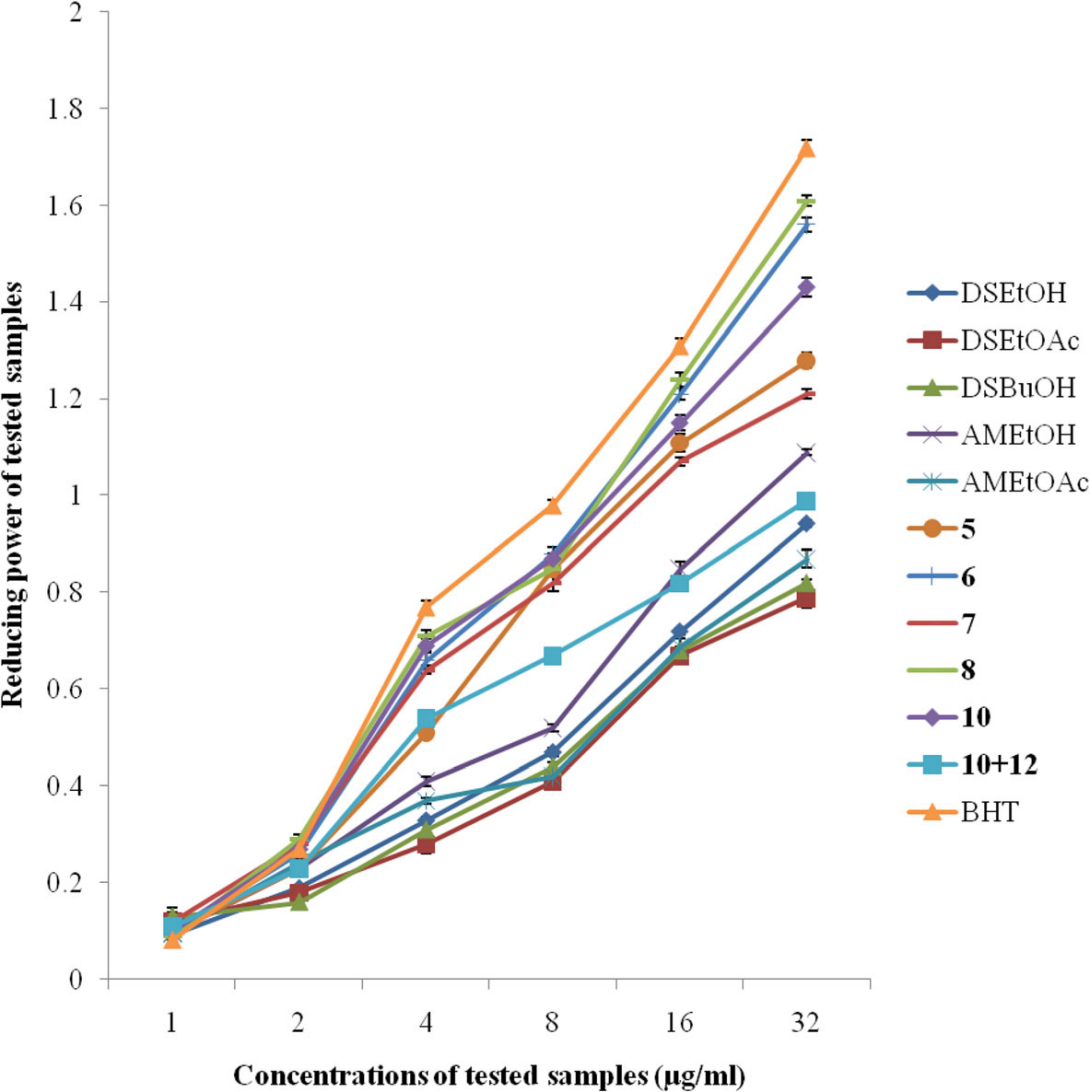
Reducing power activities of the tested samples as well as butylated hydroxytoluene (BHT). Results represent the mean ± standard deviation of the triplicate reducing power at each concentration. Compounds **1–2**, **11** and **14** were not tested; compounds **3–4**, **9** and **13** were not active; DSEtOH = *D. senegambiensis* EtOH extract; DSEtOAc = *D. senegambiensis* EtOAc extract; DSBuOH = *D. senegambiensis n*-BuOH extract; AMEtOH = *A. monticola* EtOH extract; AMEtOAc = *A. monticola* EtOAc extract

In this study, all the investigated samples showed concentration-dependent reducing power (Fig. 2). The EtOH extracts from *D. senegambiensis* and *A. monticola* displayed the largest reductive abilities when compared with their fractions. Interestingly, compounds **7** and **10 + 12** showed the lowest reducing power whereas compound **8** exhibited the highest reducing power at the different concentrations tested. The antioxidant power of compound **8** is almost equal to that of butylated hydroxytoluene (BHT) used as standard antioxidant.

### DPPH free radical scavenging activity

**Fig. 3:**
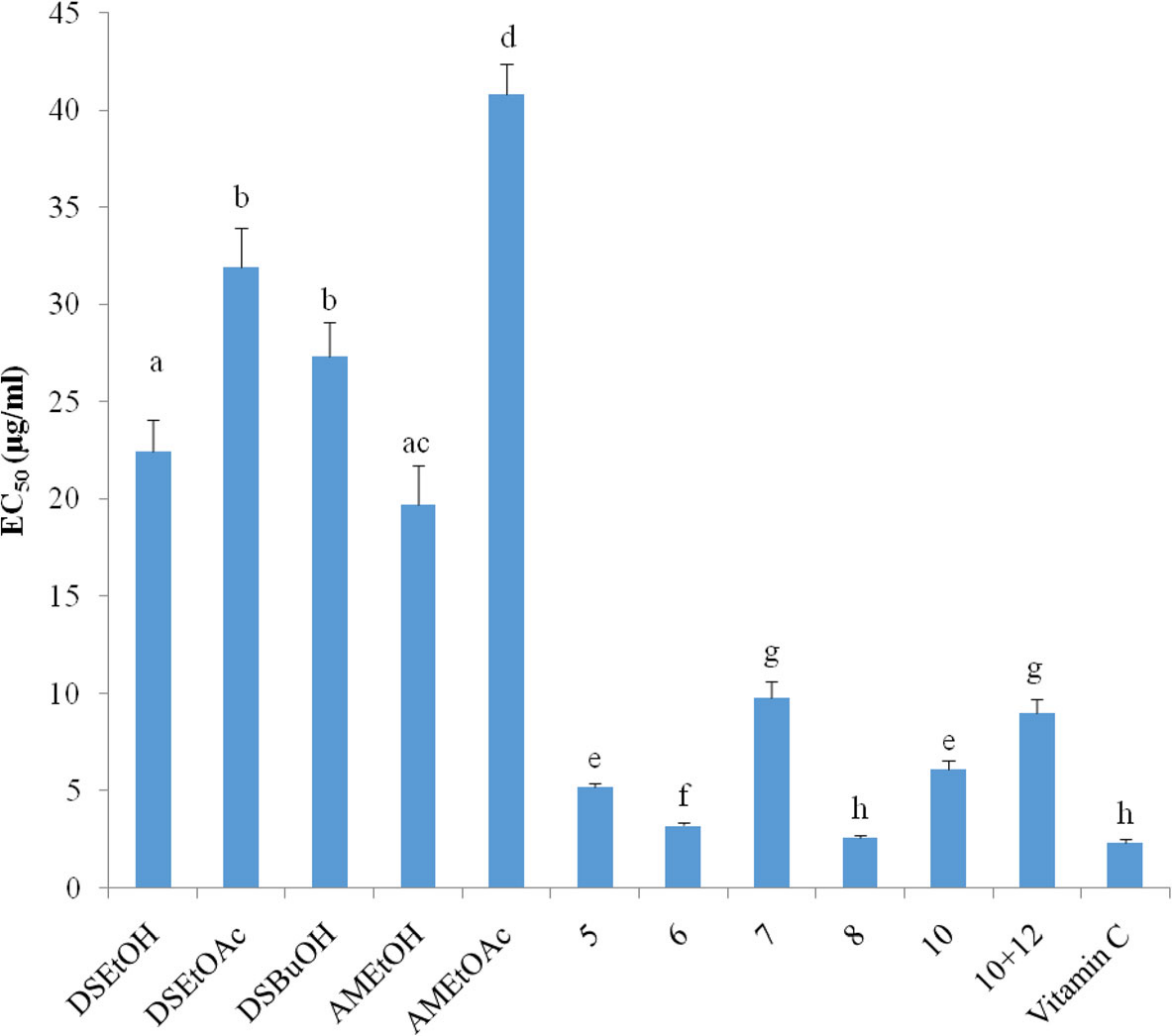
Equivalent concentrations of tested samples scavenging 50% of DPPH radical (EC_50_). Results represent the mean ± standard deviation of the triplicate EC_50_ of each sample. Letters a - h indicate significant differences between samples according to one way ANOVA and Waller Duncan test; *p* < 0.05. Compounds **1–2**, **11** and **14** were not tested; compounds **3–4**, **9** and **13** were not active; DSEtOH = *D. senegambiensis* EtOH extract; DSEtOAc = *D. senegambiensis* EtOAc extract; DSBuOH = *D. senegambiensis n*-BuOH extract; AMEtOH = *A. monticola* EtOH extract; AMEtOAc = *A. monticola* EtOAc extract

The results of the radical-scavenging activity showed that compounds **7** and **10 + 12** had the highest EC_50_ (i.e. the lowest activity) while compound **8** had the lowest EC_50_ (i.e. the highest activity) (Fig. 3). Among the extracts, *A. monticola* EtOAc extract (EC_50_ = 40.83 ± 1.57 μg/ml) displayed the lowest activity whereas *D. senegambiensis* and *A. monticola* EtOH extracts had the highest activity (EC_50_ = 22.48 ± 1.62 and 19.74 ± 1.98 μg/ml). The DPPH free radical scavenging activity of compound **8** was comparable to that of the standard antioxidant vitamin C. These results corroborate the FRAP assay, where this compound exhibited the best antioxidant activity.

### Hemolytic activity

To investigate the potential use of extracts and compounds **1**–**14**, the cytotoxicity also has to be evaluated. In this study, none of the tested samples showed hemolytic activities against red blood cells at concentrations up to 256 μg/mL and 2048 μg/mL for isolated compounds and extracts respectively (results not shown). This finding highlights the fact that the observed biological activity is not due to cellular toxicity.

## Discussion

The findings of the present study showed that there were differences between the antimicrobial activities of plant extracts. These differences may be due to the different groups of secondary metabolites found in these extracts. Indeed, the antimicrobial activity of medicinal plants is correlated with the presence in their extracts of one or more classes of bioactive secondary metabolites [39]. The results also showed that the fractionation of EtOH extracts of *D. senegambiensis* and *A. monticola* reduced their antimicrobial activity in EtOAc and *n*-BuOH extracts. This indicates that the active principles might be more concentrated in the EtOH extracts and more diluted in their fractions. The antimicrobial activity of plant extracts is considered to be highly active if the MIC < 100 μg/mL; significantly active when 100 ≤ MIC ≤512 μg/mL; moderately active when 512 < MIC ≤2048 μg/mL; weakly active if MIC > 2048 μg/mL and not active when MIC > 10 mg/mL [40]. Hence, the EtOH extract of *D. senegambiensis* was highly active (MIC < 100 μg/mL) against *S. aureus* MSSA1; significantly active (100 ≤ MIC ≤512 μg/mL) against *S. aureus* ATCC25923, *S. aureus* MRSA3, *S. aureus* MRSA4 and *C. neoformans*; moderately active (512 < MIC ≤2048 μg/mL) on *C. albicans* and *C. tropicalis*. The antibacterial and antifungal activities of extracts support the use of *D. senegambiensis* and *A. monticola* in traditional medicine for the treatment of microbial infections.

Antimicrobial cutoff points have been defined in the literature to enable the understanding of the potential of pure compounds as follows: highly active: MIC below 1 μg/mL (or 2.5 μM), significantly active: 1 ≤ MIC ≤10 μg/mL (or 2.5 ≤ MIC < 25 μM), moderately active: 10 < MIC ≤100 μg/mL (or 25 < MIC ≤250 μM), low activity: 100 < MIC ≤1000 μg/mL (or 250 < MIC ≤2500 μM and not active: MIC > 1000 μg/mL (or > 2500 μM) [40]. Based on this, most of the antimicrobial activities of the tested triterpenoid and phenolic derivatives could be considered as significant, moderate and weak depending on the sensitive microorganisms.

As mentioned previously, triterpenes are known to display significant antimicrobial properties [41–43]. With this in mind, we examined the inhibitory activity of compounds **3**, **4**, **9** and **13** against *S. aureus* and yeast strains. Although the isolated triterpenoid derivatives did not display any significant antimicrobial activity, these compounds showed some moderate and weak anti-staphylococcal activity as well as weak antifungal activity against *C. albicans*, *C. tropicalis* and *C. neoformans*. Generally, compounds **7**, **10** and the mixture of **10** + **12** showed prominent activity against methicillin-resistant *S. aureus* MRSA3 and MRSA4 and other microbes. Although the test compounds were not as active as the standard drugs, ciproflaxacin and nystatin, these compounds may be employed in situations where there is resistance to anti-staphylococcal drugs. Compounds **7** and **10** are therefore the lead candidates in the search for antimicrobial agents.

From the structure-activity-relationship point of view, compounds **4**, **5** and **6** with the same basic skeleton, have the sugar moieties which should be responsible for the differences in their activity. The difference in the antimicrobial activity of compounds **7** and **10** suggests that the contribution of electron-donating groups (-OH and –OCH_3_) is remarkable in influencing the activity. The antimicrobial activities of purified phenolic derivatives corroborate with those of the early reports against bacteria and fungi [5, 26, 44]. The antimicrobial inhibitory mechanisms of phenolic compounds found active in this study, may be due to iron deprivation or hydrogen bounding with vital proteins such as microbial enzymes [45]. Lipophilic flavonoids may disrupt microbial membranes whereas terpenes may have the ability to disrupt microbial membrane and this may explain their antimicrobial properties [46].

Reducing power is associated with antioxidant activity and may serve as a significant reflection of the antioxidant activity [47]. In this study, the crude extracts, fractions and isolated compounds from *D. senegambiensis* and *A. monticola* exhibited concentration-dependent reducing power. The reducing capacity of extracts is much related to the presence of biologically active compounds (phenols) with potent donating abilities [48]. The antioxidant potential of each extract/compound was also measured using the change in its absorbance of decolourized DPPH free-radical as it accepts electrons from the antioxidant-rich samples. A free radical is a species capable of independent existence that contains one or more unpaired electrons. Free radicals contribute to the elimination of infected cells, but they can also react with cellular DNA or other macromolecules, either damaging them directly or setting in motion a chain reaction resulting in extensive damage of cellular structures [49]. The present study showed that the free radical scavenging activity of *D. senegambiensis* and *A. monticola* is due to the presence of antioxidant-rich compounds like phenolic derivatives. Indeed, phenolic compounds are known to be potential antioxidants due to their ability to scavenge free radicals and active oxygen species such as singlet oxygen, superoxide anion and hydroxyl radicals [50]. Hence, the presence of such compounds could explain the antioxidant activity found in the studied plant extracts. The results of the antioxidant study show that extracts from *D. senegambiensis* and *A. monticola* as well as compounds **5–8, 10** and mixture of **10 + 12** may have great relevance in the prevention and therapies of diseases in which oxidants or free radicals are implicated.

## Conclusions

The phytochemical study of the EtOH extracts from the studied plant species afforded fourteen triterpenoid and phenolic derivatives. Compounds obtained from *D. senegambiensis* are *β*-amyrin palmitate (**1**), *α*-amyrin acetate (**2**), ursolic acid (**3**), sitosterol-3-O-*β*-D-glucopyranoside (**4**); vitexin (**5**) and *trans*-tiliroside (**6**). Ursolic acid (**3**), sitosterol-3-O-*β*-D-glucopyranoside (**4**), 3,4′-di-*O*-methylellagic acid (**7**), dimethyl 4,4′,5,5′,6,6′-hexahydroxybiphenyl-2,2′-dicarboxylate (**8**), lupeol (**9**), ellagic acid (**10**), 3-hydroxy-4,5-dimethoxybenzoic acid (**11**), 3-*O*-methylellagic acid 4′-*O*-*β*-D-xylopyranoside (**12**), oleanolic acid (**13**), and amphiblemmone A (**14**) were isolated from *A. monticola*. The present study revealed the potential of *D. senegambiensis* and *A. monticola* as well as the most active compounds (**7, 8** and **10)** in the search for new antimicrobial and antioxidant agents. So, further investigations are needed.

## Additional files

Additional file 1: NMR and Mass spectra of isolated compounds from *D. senegambiensis* and *A. monticola.* (PDF 1113 kb)

## Abbreviations

^*13*^*C-NMR*: Carbon thirteen Nuclear Magnetic Resonance
^*1*^*H NMR*: Proton Nuclear Magnetic Resonance
*AMEtOAc*: *A. monticola* EtOAc extract
*AMEtOH*: *A. monticola* EtOH extract
*ATCC*: American Type Culture Collection
*CC*: column chromatography
*DMSO*: Dimethylsulfoxide
*DSBuOH*: *D. senegambiensis n*-BuOH extract
*DSEtOAc*: *D. senegambiensis* EtOAc extract
*DSEtOH*: *D. senegambiensis* EtOH extract
*EtOAc*: Ethyl acetate
*EtOH*: Ethanol
*HNC*: Herbier National du Cameroun
*IR*: Infra-red
*MBC*: Minimum bactericidal concentration
*MFC*: Minimum fungicidal concentration
*MHA*: Mueller Hinton agar
*MHB*: Mueller Hinton broth
*MIC*: Minimum inhibitory concentration
*MMC*: Minimum Microbicidal Concentrations
*MS*: Mass Spectrometry
*NA*: Nutrient agar
*n*-*BuOH*: *n*-Butanol
*NMR*: Nuclear Magnetic Resonance
*SDA*: Sabouraud Dextrose Agar
*SDB*: Sabouraud Dextrose Broth
*SRF/CAM*: Section de réserve forestière du Cameroun
*TLC*: Thin Layer Chromatography
*UV*: Ultra-violet
