# Supplementary material for: Antimicrobial and antioxidant activities of triterpenoid and phenolic derivatives from two Cameroonian Melastomataceae plants: Dissotis senegambiensis and Amphiblemma monticola
Source: BMC Complement Altern Med. 2018 May 16;18:159. doi: 10.1186/s12906-018-2229-2 (PMC5956543; doi:10.1186/s12906-018-2229-2)
Supplement: Supplementary file 1 — NMR and Mass spectra of isolated compounds from D. senegambiensis and A. monticola. (PDF 1113 kb) [file 12906_2018_2229_MOESM1_ESM.pdf]

Antimicrobial and antioxidant activities of triterpenoid and phenolic derivatives from two Cameroonian Melastomataceae plants: *Dissotis senegambiensis* and *Amphiblemma monticola*

SUPPORTING INFORMATION

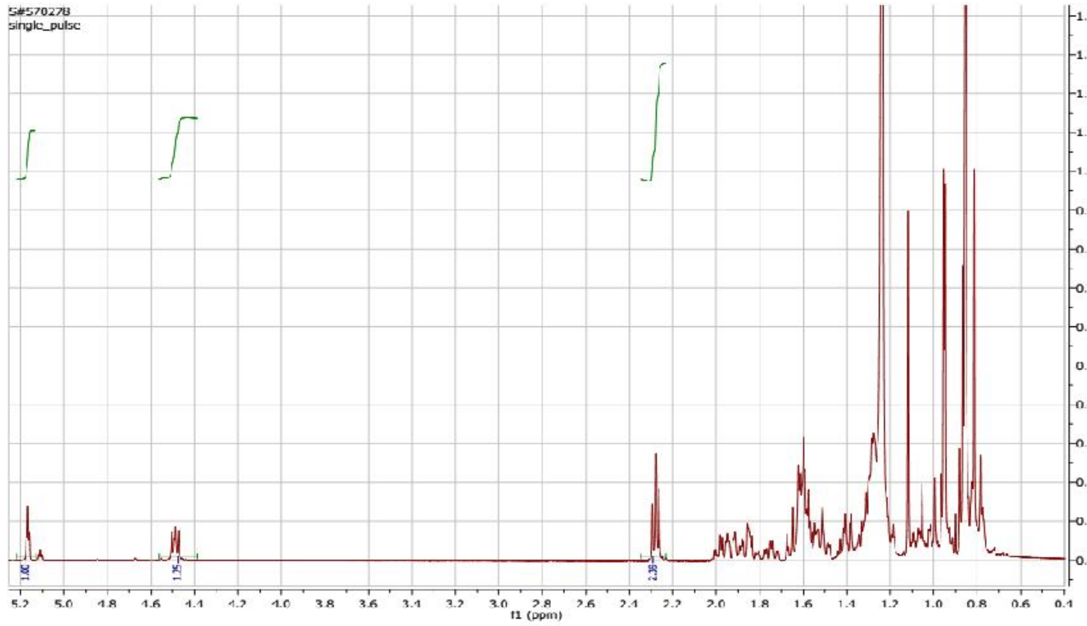

Fig. S1a ^{1}H NMR spectrum of β-amyrin palmitate (**1**; $CDCl_3$; 500 MHz)

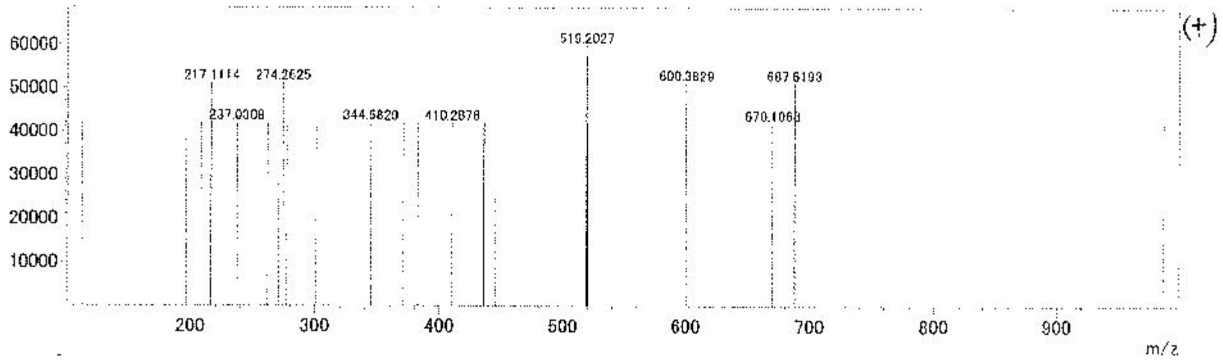

Fig. S1b HRESIMS spectrum of β-amyrin palmitate (**1**; $[(M+Na)]^+$, m/z 687.6193)

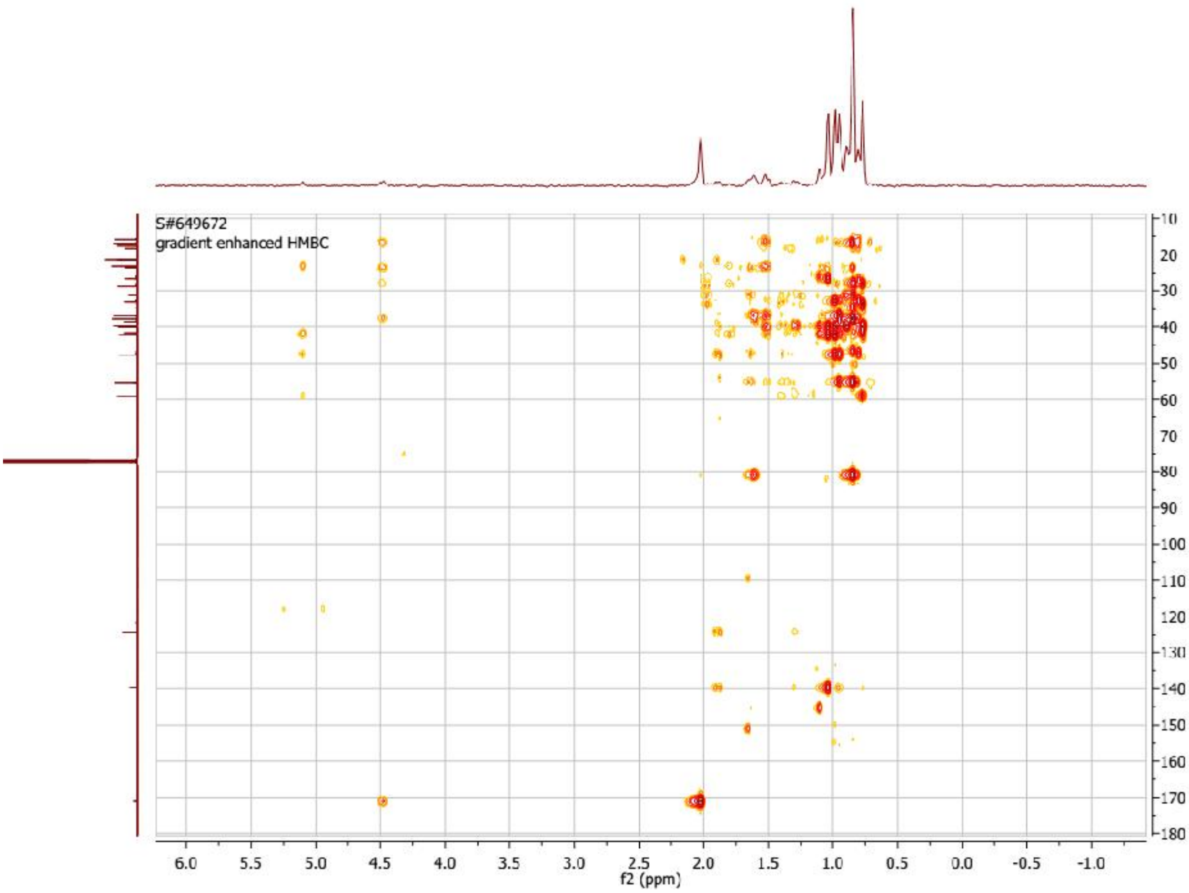

Fig. S2 HMBC spectrum of α-amyrin acetate (**2**; $CDCl_3$; 500 MHz) (showing the ^{1}H- and ^{13}C- NMR spectra of **2**)

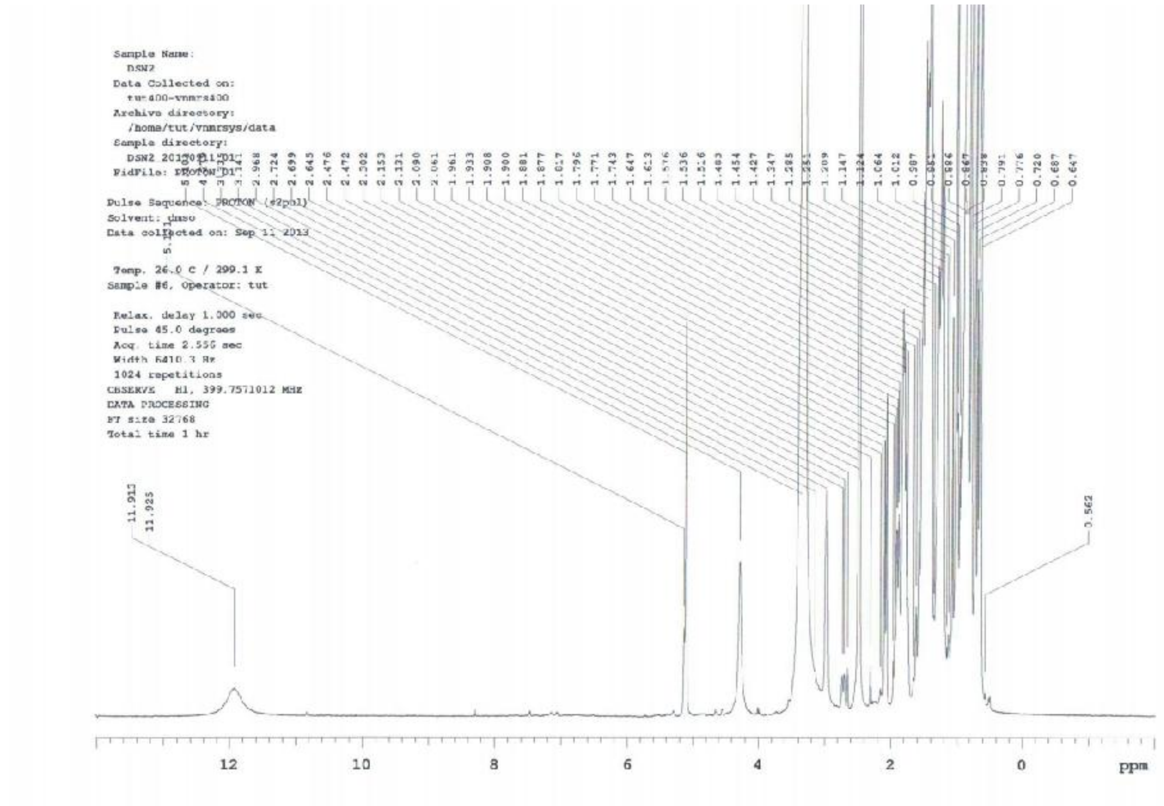

Fig. S3a ^{1}H NMR spectrum of ursolic acid (**3**; DMSO-d_6 ; 400 MHz)

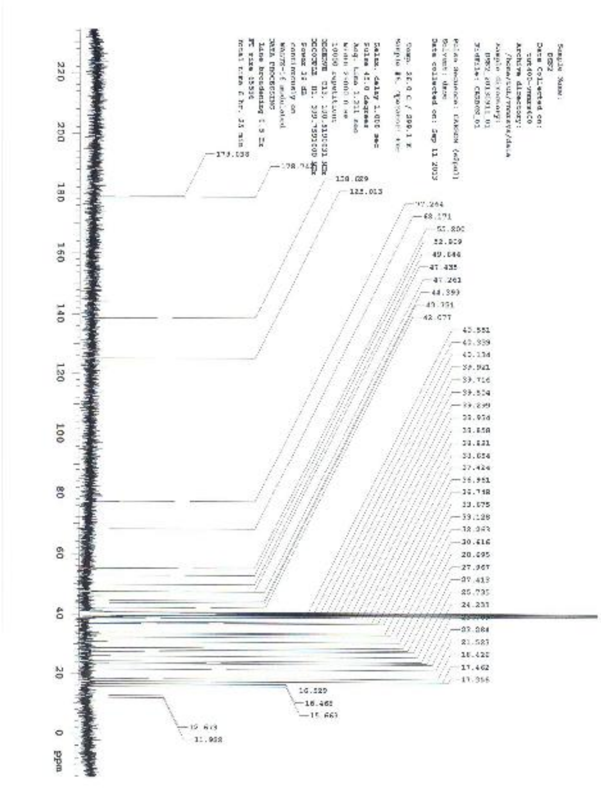

Fig. S3b ^{13}C NMR spectrum of ursolic acid (**3**; DMSO-d_6 ; 400 MHz)

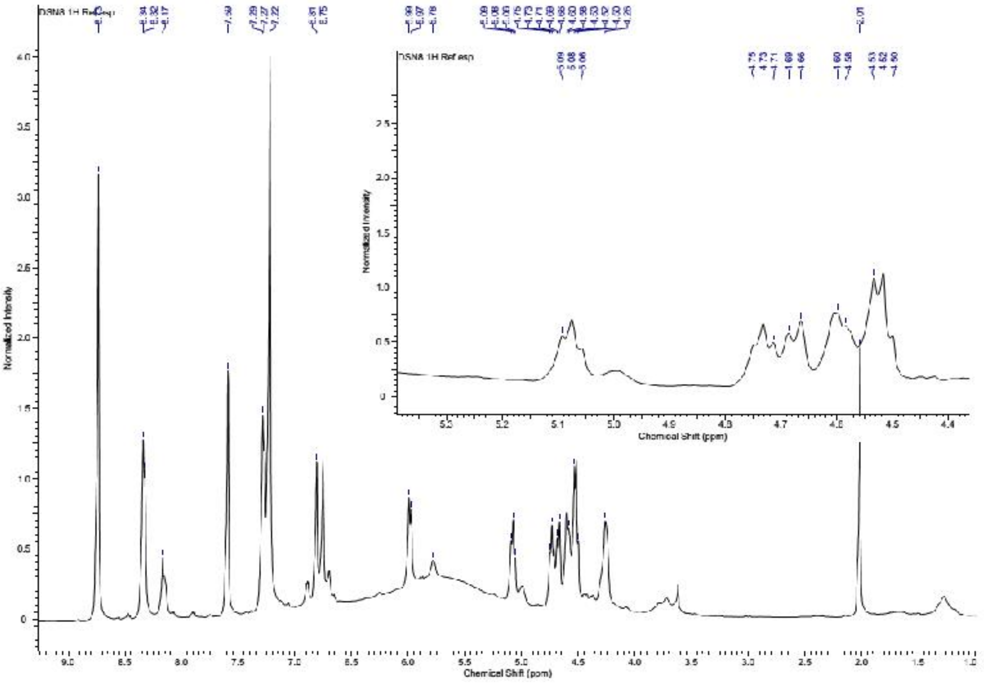

Fig. S4a ^{1}H NMR spectrum of vitexin (**5**; C_5D_5N; 500 MHz)

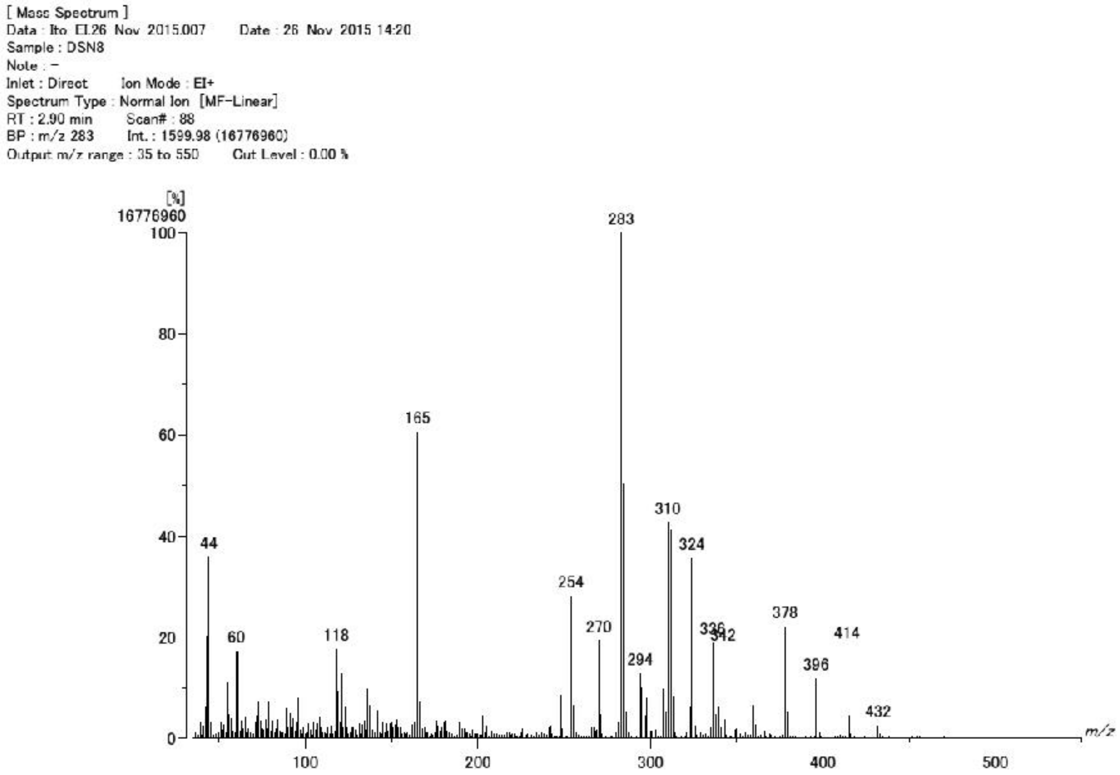

Fig. S4b EIMS spectrum of vitexin (**5**; M^+ at m/z 432)

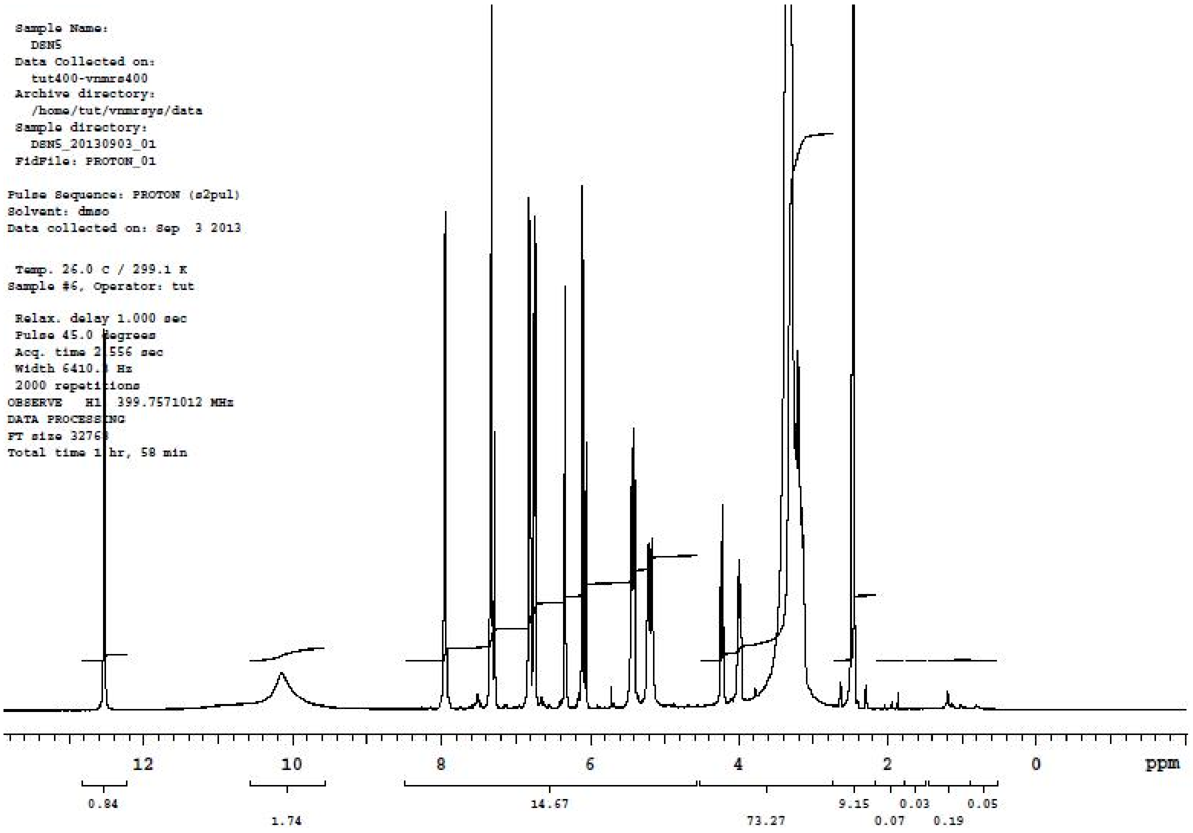

Fig. S5a ^{1}H NMR spectrum of *trans*-tiliroside (**6**; DMSO-d_6 ; 400 MHz)

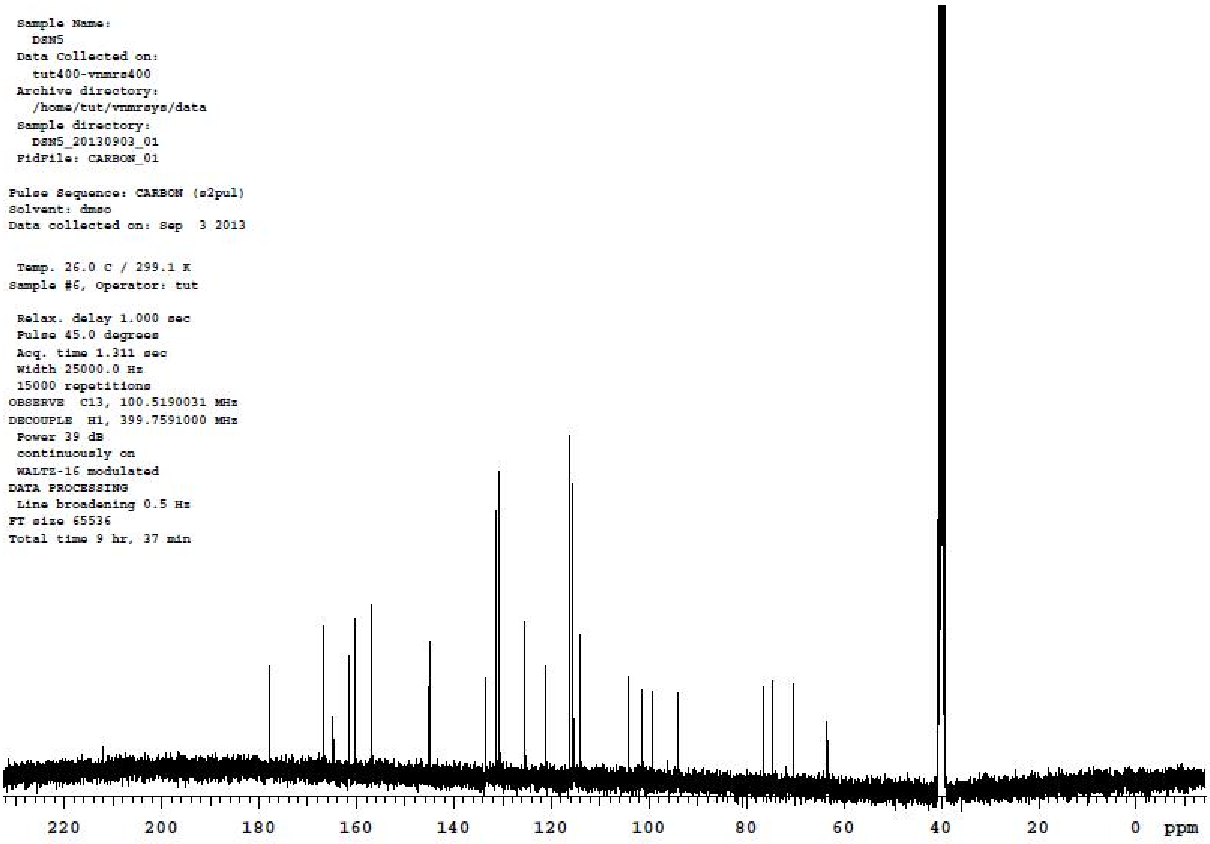

Fig. S5b ^{13}C NMR spectrum of *trans*-tiliroside (**6**; DMSO-d_6 ; 100 MHz)

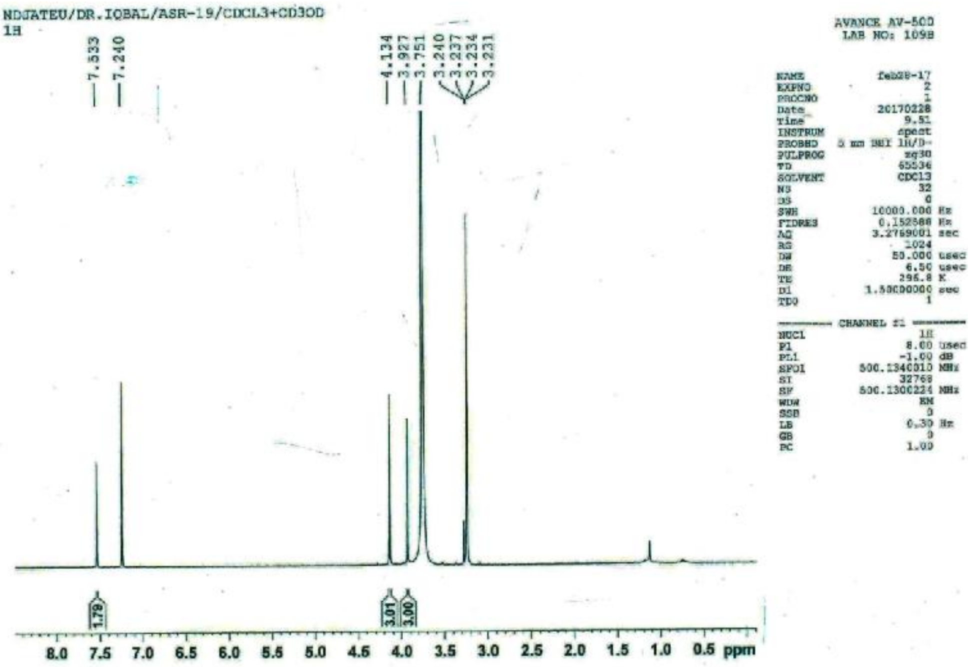

Fig. S6a ^{1}H NMR spectrum of 3,4'-di-*O*-methylellagic acid (**7**; $CDCl_3$; 400 MHz)

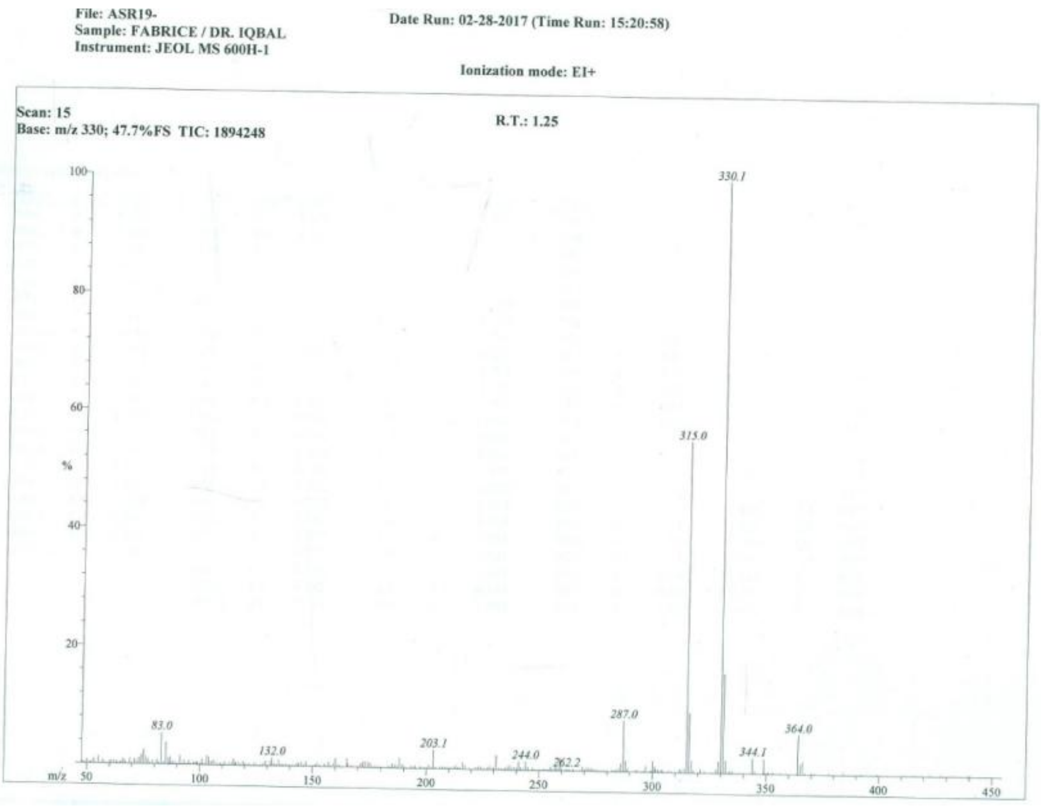

Fig. S6b EIMS spectrum of 3,4'-di-*O*-methylellagic acid (**7**; M+ at m/z 330)

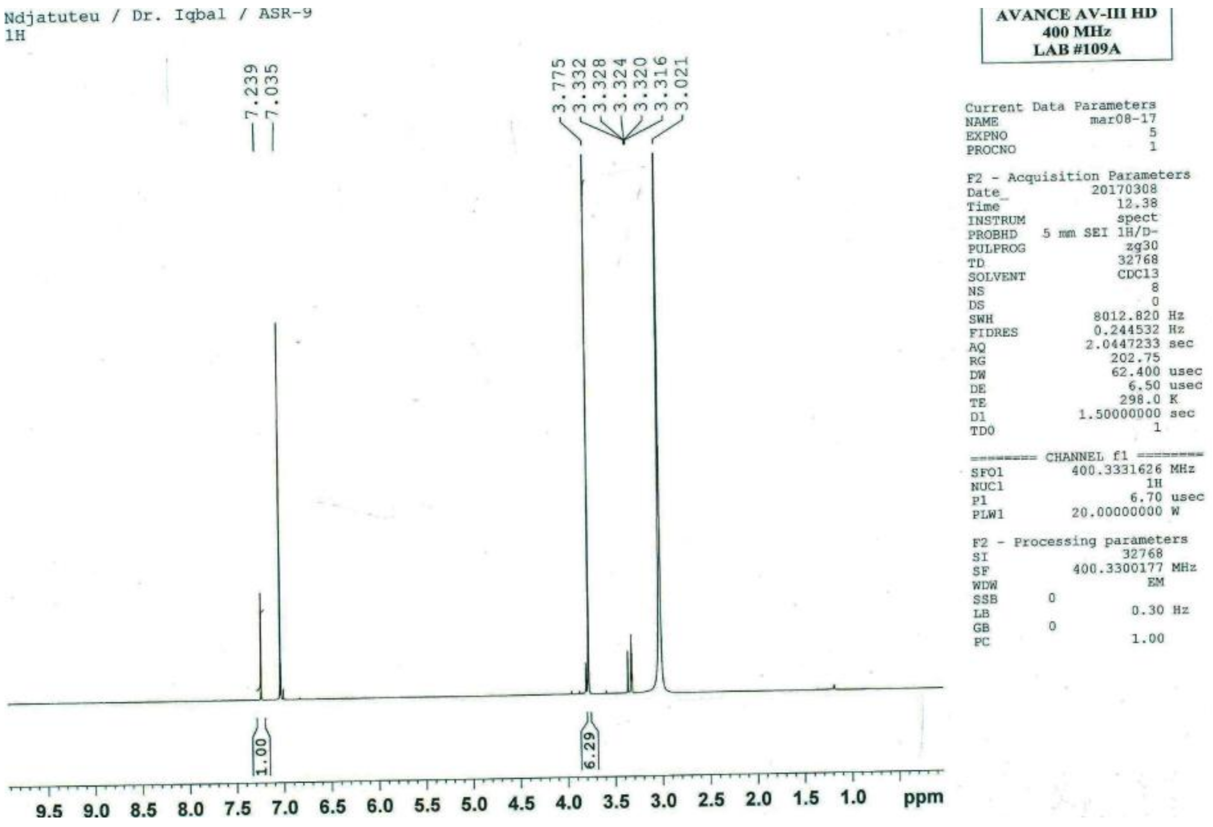

Fig. S7a ^{1}H NMR spectrum of dimethyl 4,4',5,5',6,6'-hexahydroxybiphenyl-2,2'-dicarboxylate (**8**; $CDCl_3$ + traces MeOH-*d4*; 400 MHz)

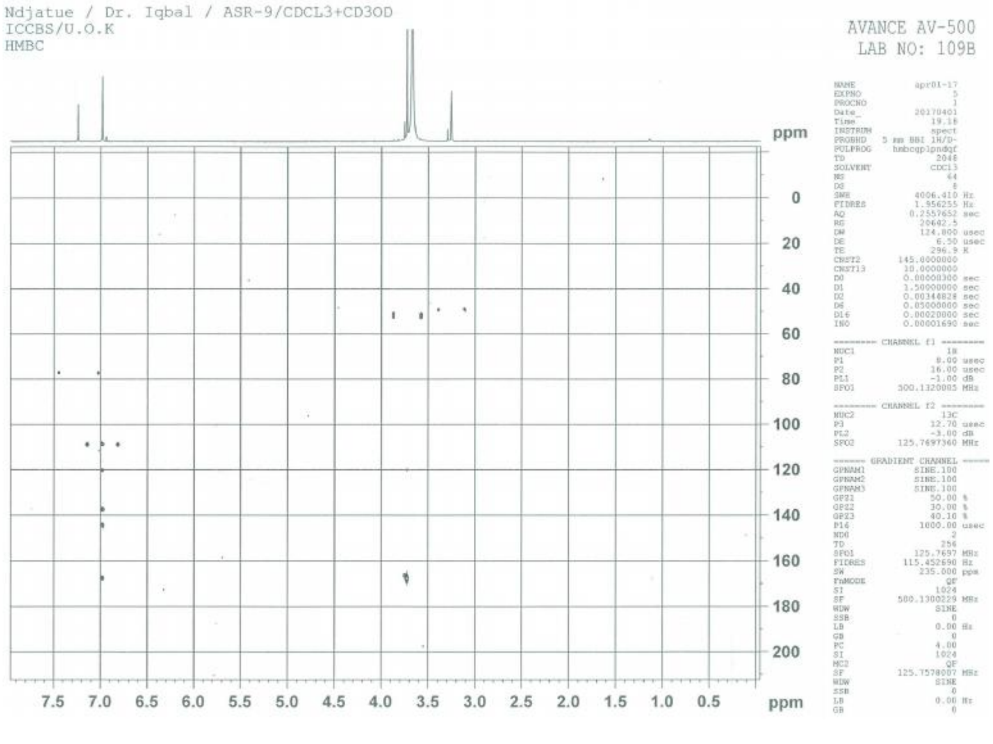

Fig. S7b HMBC spectrum of dimethyl 4,4',5,5',6,6'-hexahydroxybiphenyl-2,2'-dicarboxylate (**8**; $CDCl_3$ + traces MeOH-*d4*; 500 MHz)

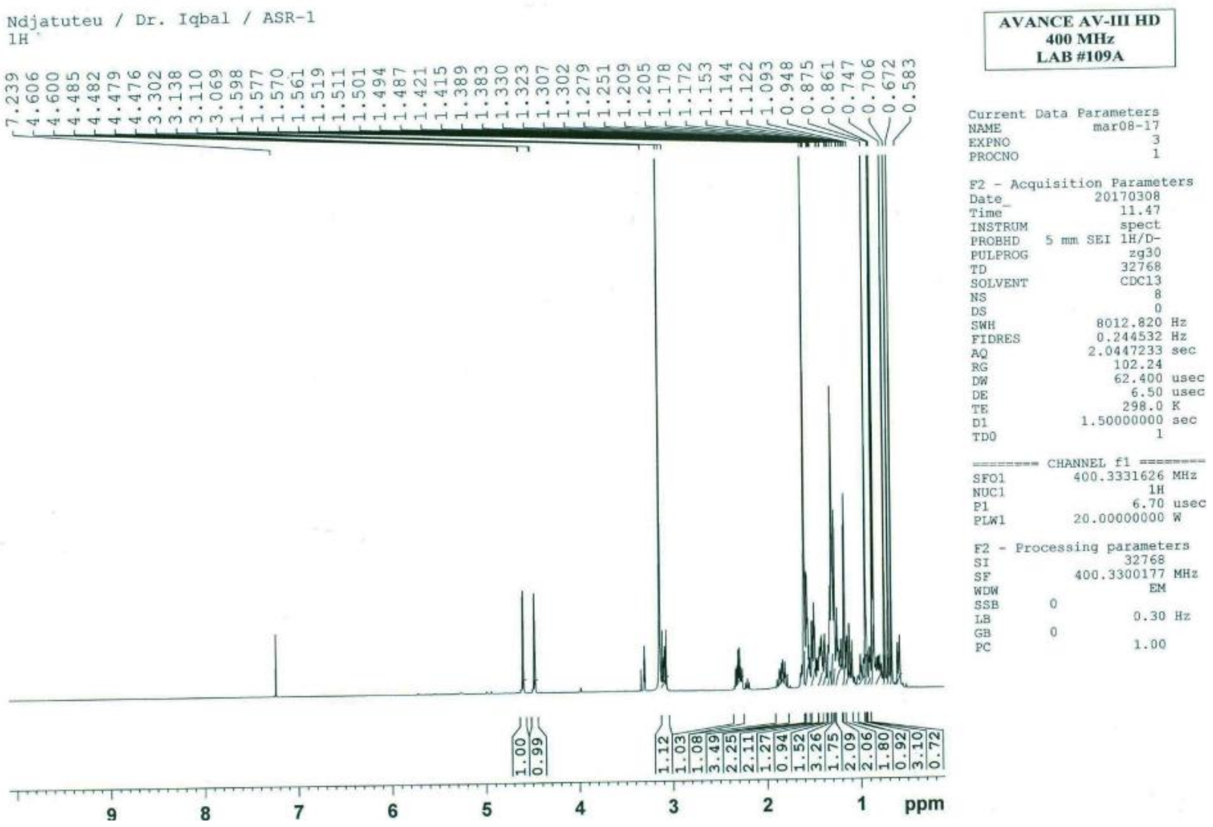

Fig. S8a ^{1}H NMR spectrum of lupeol (**9**; $CDCl_3$; 400 MHz)

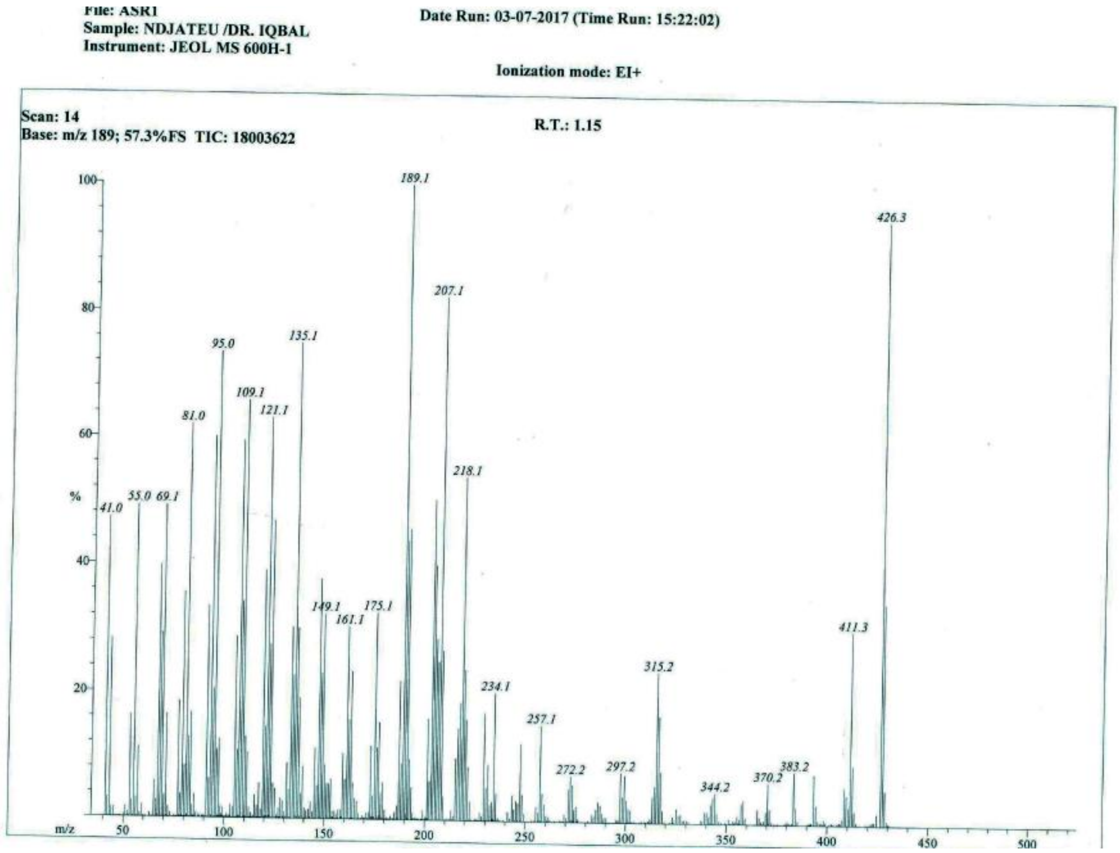

Fig. S8b EIMS spectrum of the lupeol (**9**; M^+ at m/z 426.3)

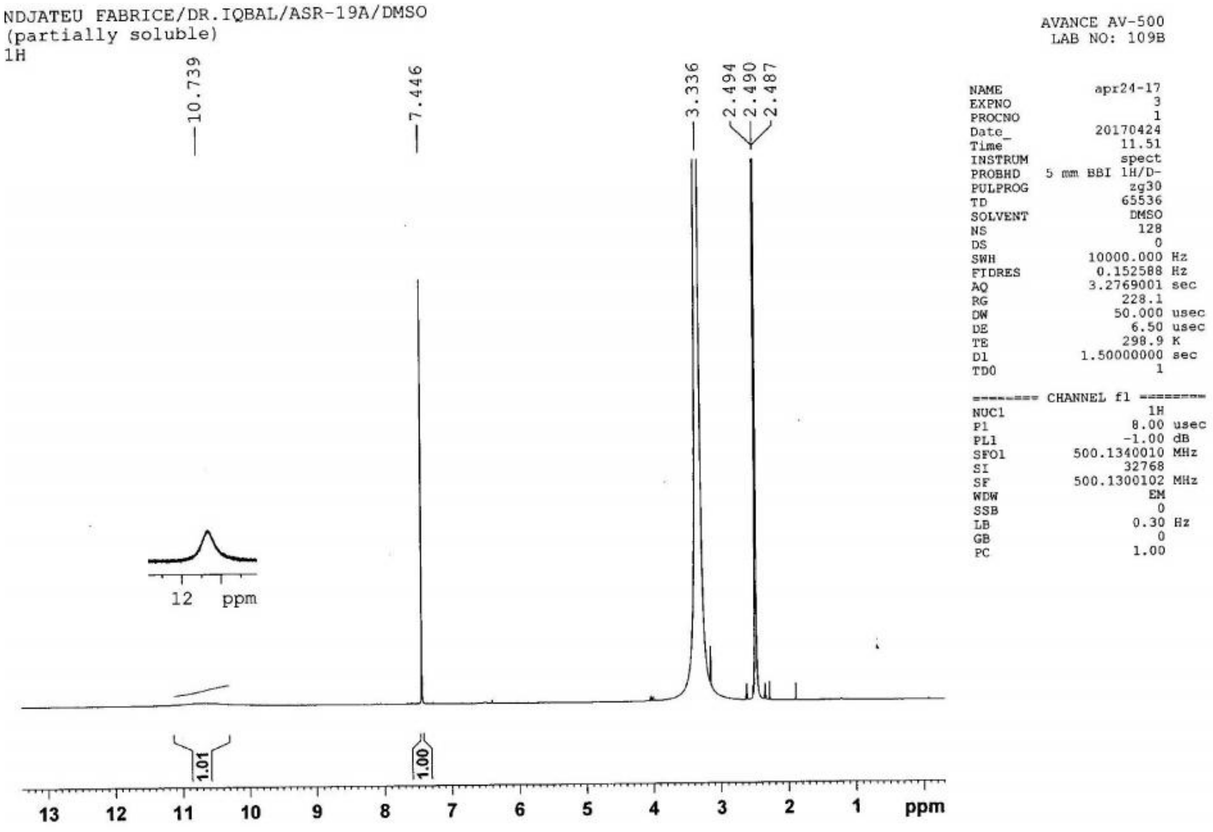

Fig. S9a ^{1}H NMR spectrum of ellagic acid (**10**; DMSO-*d6*; 500 MHz)

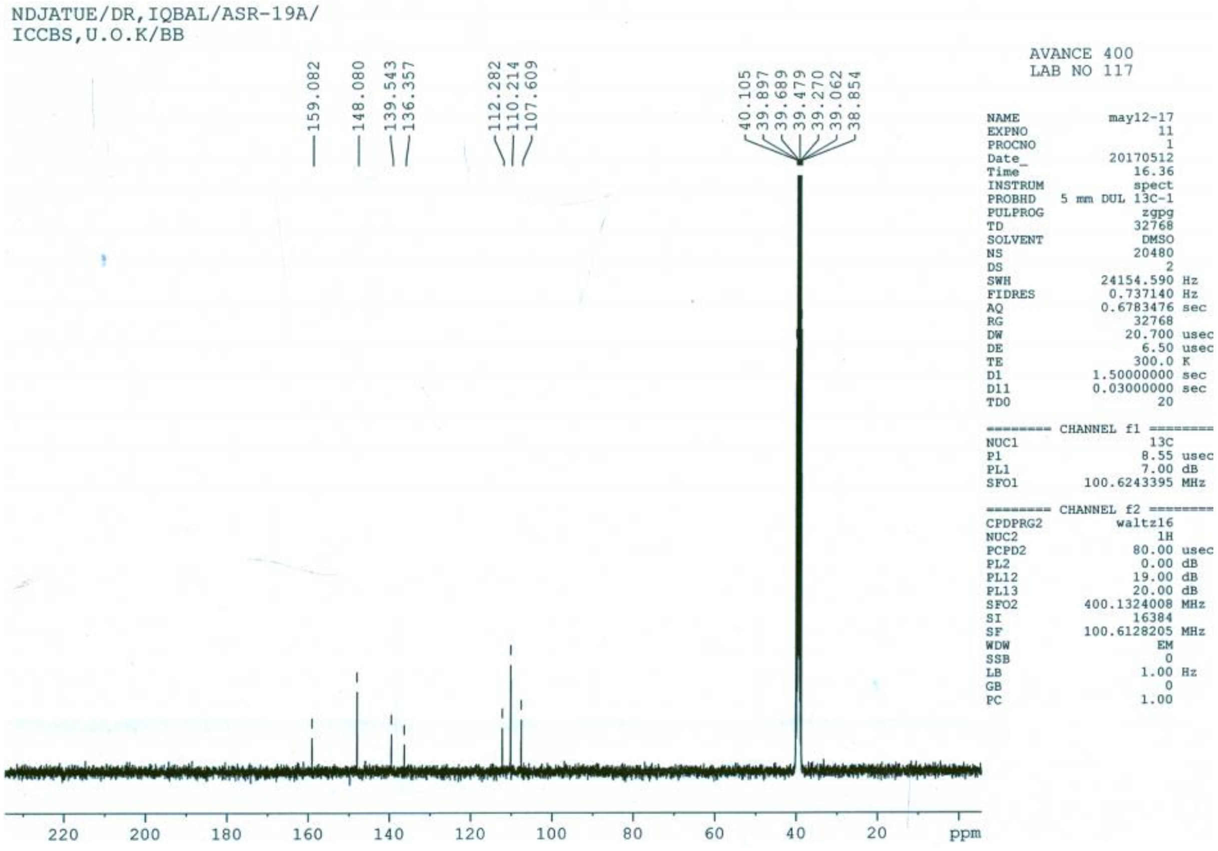

Fig. S9b ^{13}C NMR spectrum of ellagic acid (**10**; DMSO-*d6*; 125 MHz)

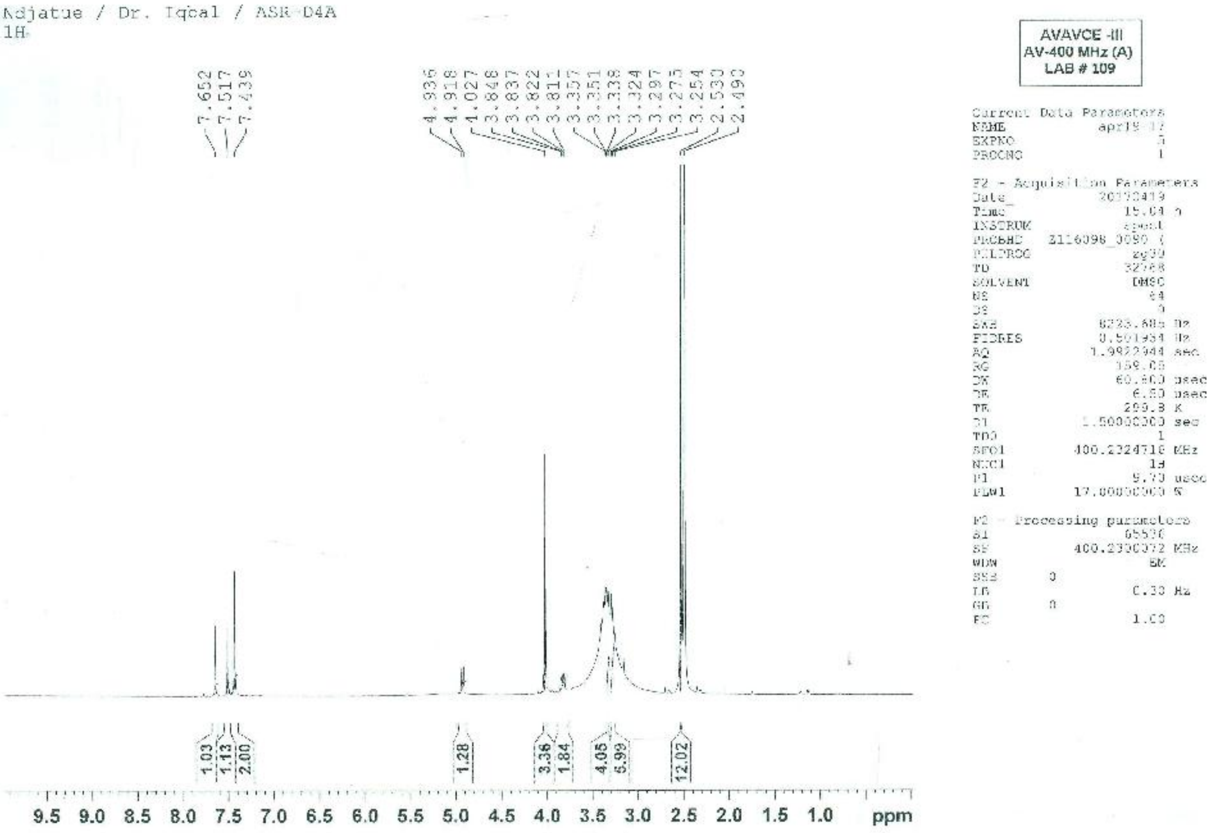

Fig. S10a ^{1}H NMR spectrum of the mixture of ellagic acid and 3-*O*-methylellagic acid 4′-*O*-*β*-D-xylopyranoside (**10 + 12**; DMSO-d_6 ; 400 MHz)

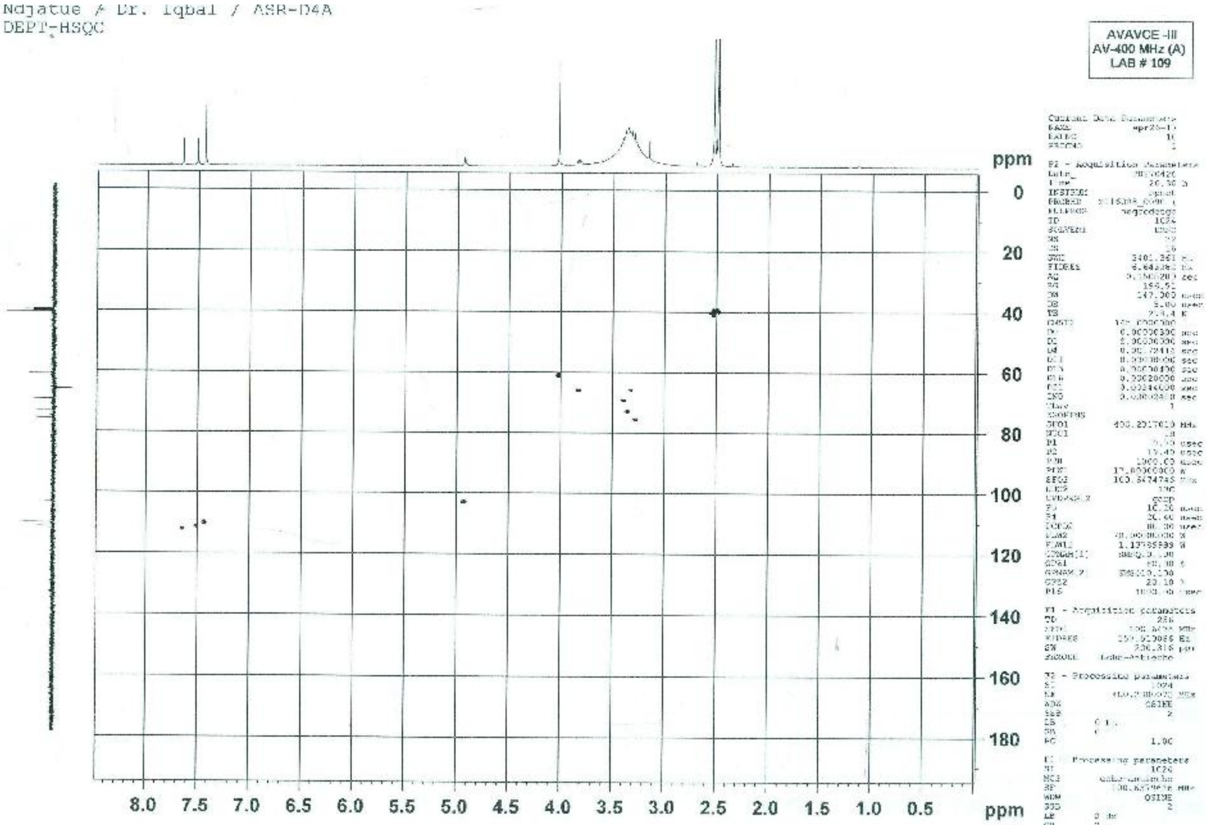

Fig. S10b HSQC spectrum of the mixture of ellagic acid and 3-*O*-methylellagic acid 4′-*O*-*β*-D-xylopyranoside (**10 + 12**; DMSO-d_6 ; 400 MHz)

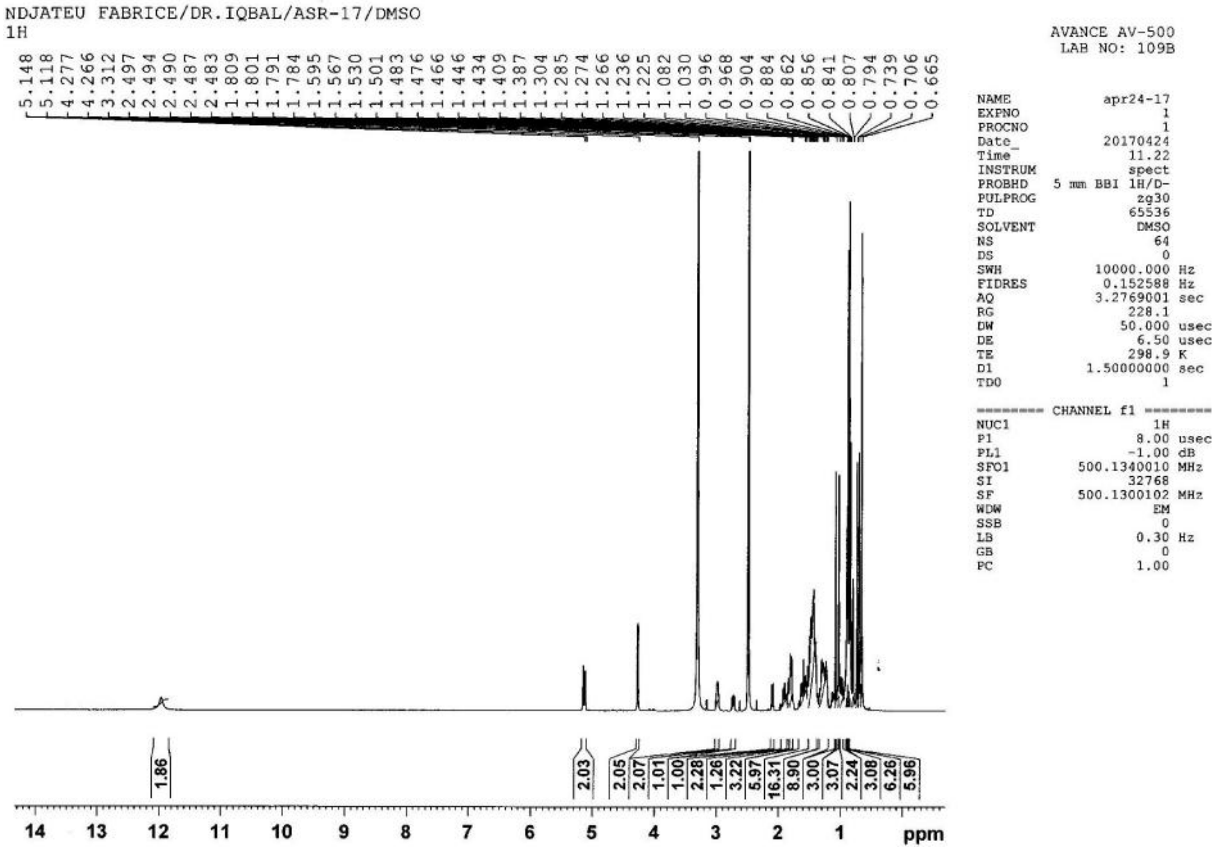

Fig. S11a ^{1}H NMR spectrum of the mixture of ursolic acid and oleanolic acid (**3 + 13**; DMSO-d_6 ; 500 MHz)

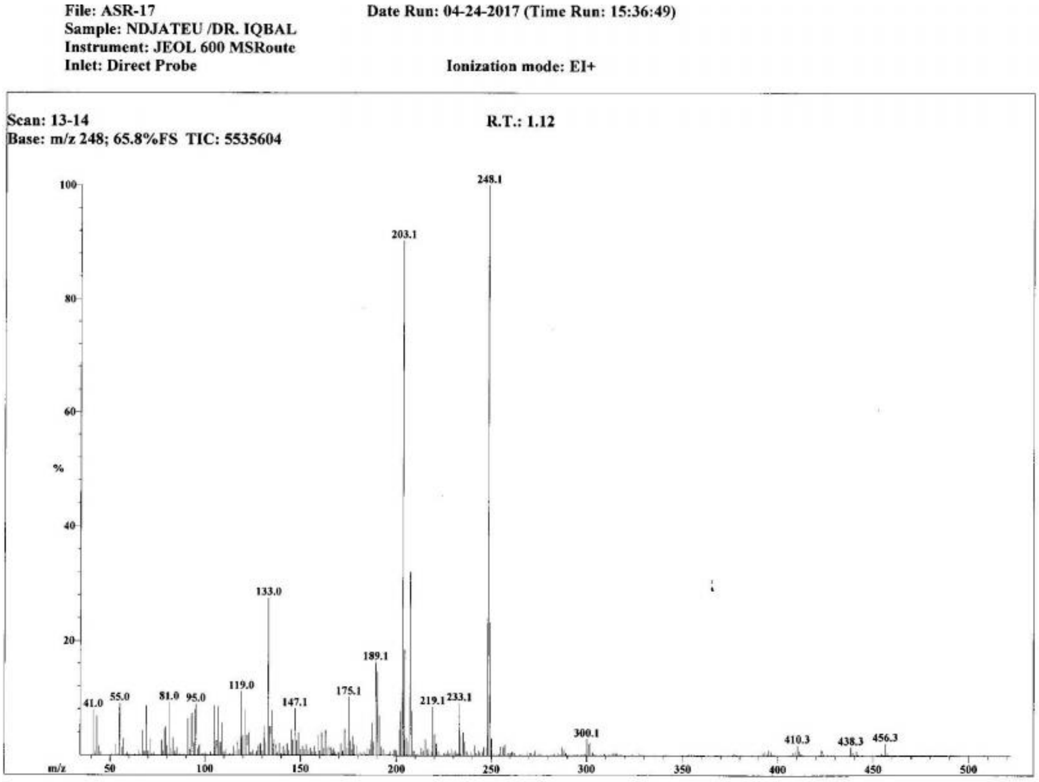

Fig. S11b EIMS spectrum of the mixture of ursolic acid and oleanolic acid (**3 + 13**; M^+ at m/z 456.3)

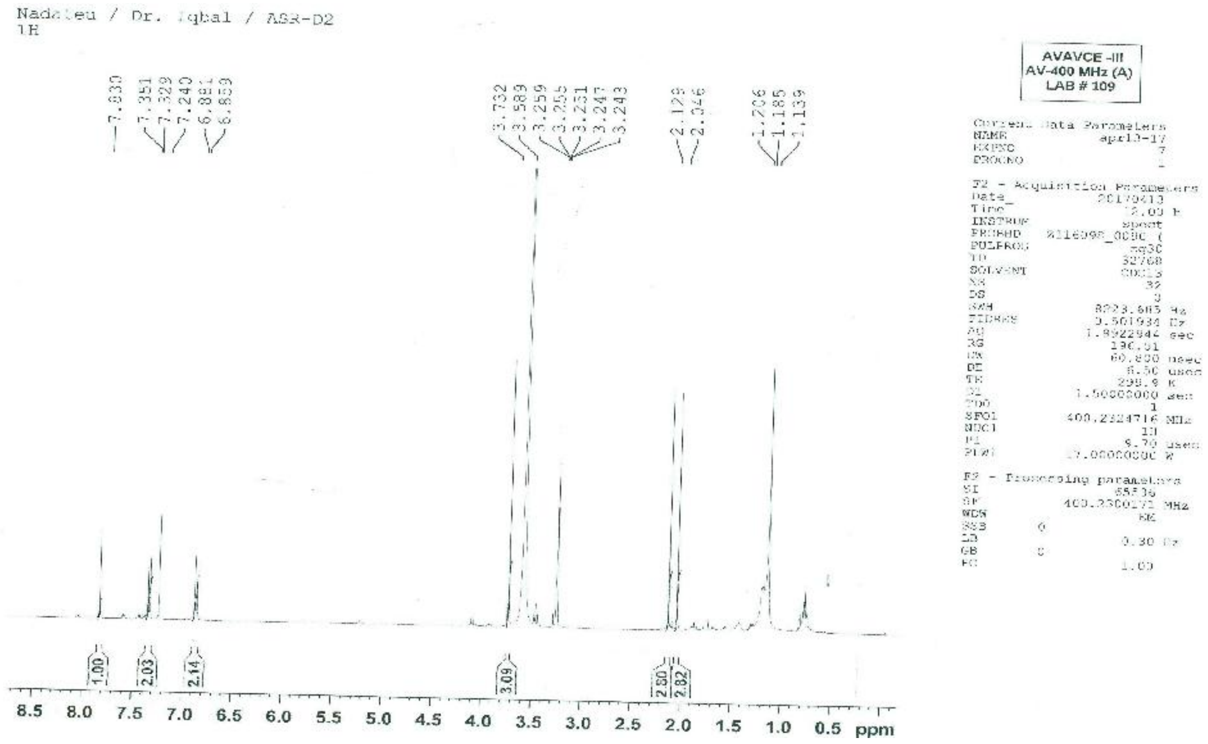

Fig. S12a ^{1}H NMR spectrum of amphiblemmone A (**14**; $CDCl_3$ +traces MeOH-$_{d4}$; 400 MHz)

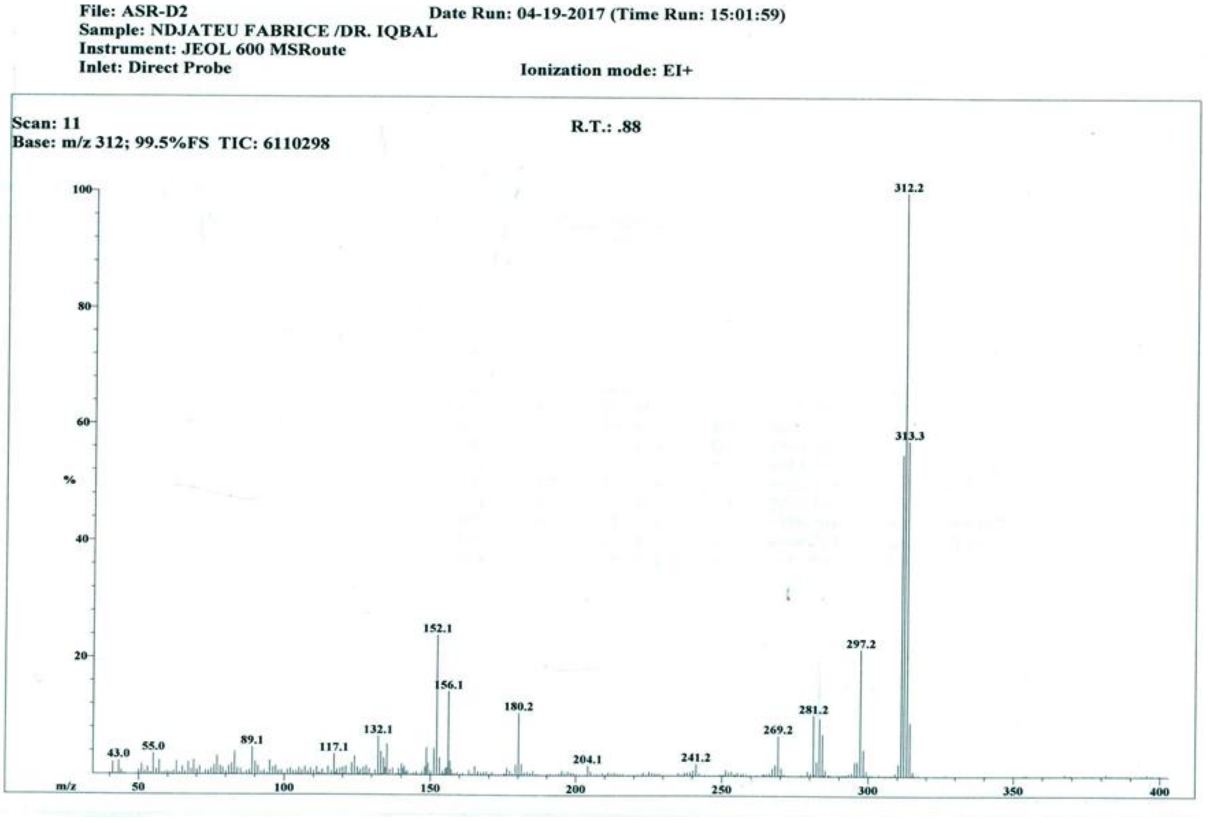

Fig. S12b EIMS spectrum of amphiblemmone A (**14**; M^+ at m/z 312.2)

Sitosterol-3-O-β-D-glucopyranoside (**4**) and 3-hydroxy-4,5-dimethoxybenzoic acid (**11**) were identified by comparison (TLC, mixed TLC) with authentic samples found in the laboratory.
